# Bioactive Indanes: Proof of Concept Study for Enantioselective Synthetic Routes to PH46A, a New Potential Anti-Inflammatory Agent

**DOI:** 10.3390/molecules23071503

**Published:** 2018-06-21

**Authors:** Tao Zhang, Gaia Scalabrino, Neil Frankish, Helen Sheridan

**Affiliations:** 1 Trino Therapeutics Ltd., The Tower, Trinity Technology and Enterprise Campus, Dublin 2, D02 W272, Ireland; zhangt@tcd.ie (T.Z.); gaiascal@gmail.com (G.S.); nfrnkish@tcd.ie (N.F.); 2 Novel Drug Discovery Group, School of Pharmacy and Pharmaceutical Sciences & Trinity Biomedical Sciences Institute, Trinity College, Dublin 2, D02 W272, Ireland

**Keywords:** bioactive indanes, PH46A, enantioselective, biocatalysis, hydrolases, phase-transfer catalyst (PTC)

## Abstract

PH46A is a single enantiomer and a member of the 1,2-indane dimer family. It has two contiguous stereogenic centers with *S*,*S* configurations, one of which being a quaternary center, which has been developed as a clinical candidate for the treatment of inflammatory and autoimmune conditions. The current synthetic route to PH46A involves the generation of an unwanted enantiomer (*R*,*R*)-**7**, thus reducing the final yield significantly. Therefore, we have investigated potential alternatives to improve the efficiency of this synthesis. The first phase of the study has demonstrated proof of principle for a chiral alkylation of ketone **3** using phase-transfer catalysis, providing a key intermediate ketone (*S*)-**4**. The parent alkaloids required for the synthesis of PH46A, quinine or cinchonidine, have also been identified. Promising enantiomeric excesses of up to 50% have been achieved to date, and the use of an alternative substrate, unsaturated ketone **9**, has also opened up further avenues for optimisation in future studies. The second part of the study involved preliminary screening the effects of a panel of hydrolase enzymes on (*rac*)-**4** in order to identify a potential chemo-enzymatic route to optimise the introduction of chirality into PH46A at early stage of the synthesis. The hydrolase module has also yielded positive results; enzyme AH-46 with MtBE providing a selectivity factor of 8.4 with enantiomeric excess of 77%. Overall, positive results were obtained in this proof of concept study described herein. It is believed that conditions of both chiral PTC alkylation and biocatalytic hydrolysis could be optimised to further enhance the selectivity and improve the overall yield. This work is currently ongoing.

## 1. Introduction

The indane scaffold is a key moiety found in a range of biologically active naturally occurring and synthetic molecules. During the course of our work, a novel indane scaffold was discovered, which demonstrated potential treatment of inflammatory conditions, in particular Inflammatory Bowel Disease (IBD) [1,2,3,4,5,6]. A lead first-in-class chiral molecule, PH46A, 6-(methylamino)hexane-1,2,3,4,5-pentanol-4-(((1*S*,2*S*)-1-hydroxy-2,3-dihydro-1*H*,1′*H*-[2,2-biinden]-2-yl)methyl)benzoate [7,8], has been shown to have a therapeutic effect in two different well-established preclinical models of murine colitis: the acute dextran sodium sulphate model and the chronic and spontaneous Interleukin-10 (IL-10^−/−^) knock-out mouse model. This is indicative that PH46A has therapeutic effect which is independent of model specific aetiology [7]. Thus, PH46A has been developed in the clinic for the treatment of IBD and it has recently completed a Phase I clinical trial study [9].

**Scheme 1:**
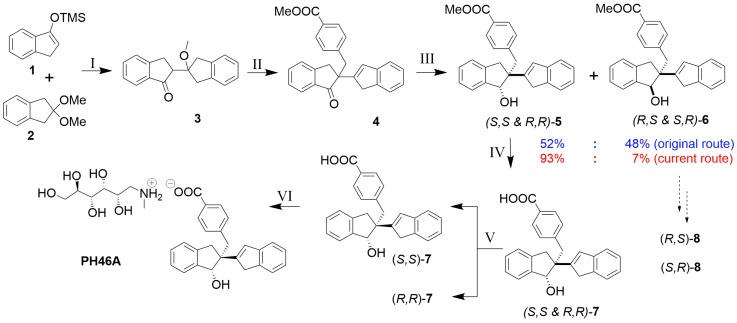
The current synthetic scheme of manufacturing PH46A [7,10]. *Reagnets and Conditions*: (I) trimethylsilyl trifluoromethanesulfonate, TEA, DCM, −70 °C to −75 °C, 3 h; (II) methyl 4-(bromomethyl)benzoate, potassium *t*-butoxide, MtBE, *t*-BuOH, room temperature, 3 h; (III) triisobutylaluminium (1.1 M) solution in toluene, toluene, −12 °C to 10 °C, 2 h; (IV) 30% NaOH, MeOH, reflux, 24 h; (V) chiral separation by simulated moving bed technology; (VI) *N*-methyl-d-glucamine, EtOH, water, 55 °C, 5 h.

The original synthetic route to PH46A involves conventional organic chemistry and enantiomeric separation achieved by preparative chiral HPLC (Scheme 1). The overall yield of the original synthetic method was considered to be less satisfactory due to the generation of an undesirable diastereoisomer hydroxyl ester racemic (*rac*)-**6** at ~48% yield and the unwanted enantiomer (*R*,*R*)-**7** at almost 50% isolation yield from its racemic mixture (*rac*)-**7** [7]. The ketone reduction step (Step III) was subsequently optimised by the use of triisobutylaluminum (TiBA) affording the desired product, diastereoisomer (*rac*)-**6**, in a good diastereoselectivity percentage (93%) [10]. Such a highly selective method has successfully been employed on multi-kilo scale in a GMP environment. However, it is recognised that the introduction of enantioselective approaches to the synthesis of PH46A would eliminate or reduce the cost burdens of generating the undesired enantiomer (*R*,*R*)-**7** and of carrying out a large scale separation of the enantiomers.

**Scheme 2:**
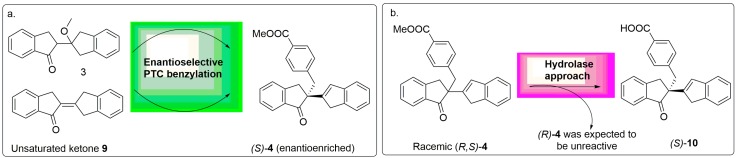
(**a**) Synthesis of (*S*)-**4** via proposed enantioselective benzylation of ketone **3** or unsaturated ketone **9**; (**b**) synthesis of (*S*)-**10** via proposed enantioselective biocatalytic hydrolysis of racemate (*R*,*S*)-**4**.

Two approaches to the optimisation of the PH46A synthesis were investigated and are presented in this paper. The first approach involved the investigation of an enantioselective synthesis of the intermediate keto methyl (Me)-ester (*S*)-**4**. In this approach the chiral phase transfer catalyst-promoted (PTC) benzylation of ketone **3** (or preferably unsaturated ketone **9**, to simplify the analysis) was identified as a particularly promising approach (Scheme 2a). Chiral alkylation using cinchona alkaloid-derived PTCs has received significant attention from an industrial perspective (largely in the synthesis of amino acids), as the conditions required are typically mild, without the requirement of exclusion of moisture or oxygen, and the catalysts are normally trivial to prepare from the parent alkaloid. The alkaloids themselves are also readily available. Of particular interest, reports from Merck [11] and Hughes et al. [12], show that 94% enantioselectivity could be achieved in alkylation using *N*-benzylcinchoninium-based catalysts in a system both chemically and sterically related to our case. This example suggested that a similar cinchonine-derived catalyst would give the desired enantiomer of (Me)-ester (*S*)-**4**, when applied to our system. However, this particular catalyst may not be suitable as the Merck researchers attributed selectivity to a π-stacking effect between the phenyl substituent of the substrate and the benzylic group in the catalyst; such an effect would be different in the extended π-system present in deprotonated unsaturated ketone **9**. As a result, an initial catalyst screen employing four cinchonine-derived catalysts under each of four reactions was planned, followed by expansion of hits in more depth. Following benzylation to give enantio-enriched (*S*)-**4**, diastereoselective reduction by TiBA can be applied to afford the final desired (*S*,*S*)-**4**.

The second approach investigated the enantioselective hydrolysis of (*rac*)-**4** using hydrolase enzymes while leaving the unfavourable enantiomer (*R*)-**4** unchanged (Scheme 2b). Hydrolase enzymes have been receiving increasing attention in the context of organic synthesis in the last decade. As biocatalysts, they provide substrate specificity with high regio- and enantio-selectivity and they enable the resolution of organic substrates with great efficiency and selectivity [13,14,15]. Lipases and proteases are the most utilised forms of hydrolytic enzymes, which demonstrate diverse substrate tolerance, stability in organic solvents and at elevated temperatures [16,17]. Several enzyme based methods of synthesising chiral alcohols have been developed and employed to date, such as enzyme-catalysed kinetic resolution of racemic substrates, asymmetric reduction of pro-chiral ketones and asymmetric hydroxylation of hydrocarbons. The scalability (up to 500 g) of some of the methods are proven [18,19,20]. It was postulated that if the proposed approach was successful, such a resolution could furnish the target keto carboxylic acid (*S*)-**10** with the desired ‘*S*′ configuration in place. This product could then be isolated and carried through into subsequent steps. It was envisaged that by screening a panel of 48 hydrolase enzymes against (*rac*)-**4**, for the transformation shown in Scheme 2b, an active and selective enzyme would be identified to afford (*S*)-**10** in high enantio-purity. As a result, we developed conditions which afforded both high selectivity and high conversion, and which are described herein.

## 2. Results and Discussion

### 2.1. Synthesis of Chiral Standards: *(*S*)-**4***, *(*R*)-**4***, *(*S*)-**10*** and *(*R*)-**10***

**Scheme 3:**
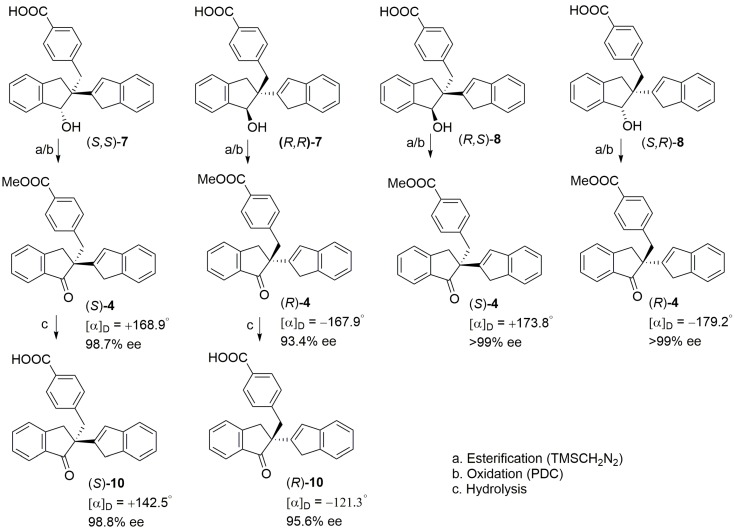
Synthetic scheme of chiral molecule standards (*S*)-**4**, (*R*)-**4**, (*S*)-**10** and (*R*)-**10**.

The syntheses of chiral standards, (*S*)-**4**, (*R*)-**4**, (*S*)-**10** and (*R*)-**10** were carried out ahead of performing designed enantiomeric selectivity screening (Scheme 3). The enantiomers (*S*,*S*)-**7**, (*R*,*R*)-**7**, (*R*,*S*)-**8** and (*S*,*R*)-**8** were obtained internally within our group, which were converted to their corresponding keto (Me)-esters **4** by esterification and oxidation. All reactions proceeded efficiently and with full conversions to yield (*S*)-**4** and (*R*)-**4**. It was found that the reactions with (*R*,*S*)-**8** and (*S*,*S*)-**7** yielded the product with a positive optical rotation value being assigned to be (*S*)-**4**, while (*S*,*R*)-**8** and (*R*,*R*)-**7** yielded the opposite enantiomer (*R*)-**4**. Following hydrolysis, keto acids (*S*)-**10** and (*R*)-**10** (with positive and negative optical rotation values, respectively) were obtained from their corresponding (Me)-esters. The enantiomeric excess (e.e.) of each product was also measured and the results are shown in Scheme 3 and Experimental section. The relevant analytical chromatograms and spectra are given in Supplementary Materials (Figures S1–S12).

### 2.2. PTC-Promoted Alkylation of Ketone ***3***

**Scheme 4:**
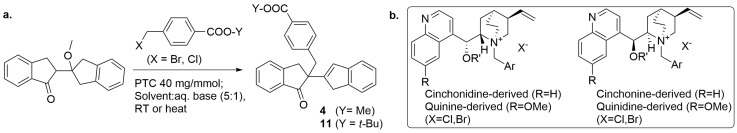
(**a**) Alkylations of ketone **3** using chiral PTCs; (**b**) general structures of cinchona alkaloids derived PTCs.

Based on the concern of the potential (Me)-ester moiety instability in methyl(4-bromomethyl)benzoate (MBMB) under PTC conditions, a more robust analogous, named *t*-butyl (*t*Bu)-(4-bromomethyl)benzoate (TBBMB), was chosen for initial testing (Scheme 4a). While a variety of parent structures for chiral PTCs are described in the literature [21,22,23,24], in practice those derived from cinchona alkaloids (Scheme 4b) are often preferred for reactions that could potentially be carried out on kilo scales. The cinchona alkaloids represent inexpensive sources of chiral information and are widely available, while the PTC derivatives themselves are generally straightforward to prepare, usually without the need for purification [25,26,27]. There are a wide variety of others available, perhaps most notably chiral crown ethers [28,29] or Maruoka’s biaryl catalysts [30,31], however, many of these would be costly to source or prepare. In our hands, a small set of PTCs derived from cinchona alkaloids was available and it was selected for the first screen, using 25% aqueous (aq.) NaOH/toluene (1:5, *v*/*v*) as for the *t*Bu-ester **11** reaction described in Scheme 4.

The details of the PTCs preparation are described in the Supplementary Materials. Most of the screening reactions were quite rapid (Table 1). However, enantioselectivity was lower than envisaged. Interestingly, Entry 1D was much slower than the others, nevertheless, it gave slightly higher enantioselectivity. The reaction rate with this particular catalyst relative to others in this small set cannot be readily correlated to selectivity, as the slower reaction in this case could simply be due to increased water solubility of the catalyst/base ion pair due to the nitro group. Subsequent investigation with the *N*-(2-NO_2_-Bn)-quinidinium bromide catalyst under a variety of conditions failed to identify any significant improvements (Entries 2A–K). Indeed, Entry 2G in dichloromethane (DCM) gave the unwanted (*S*)-enantiomer (with poor selectivity), while Entries 2F and 2H gave complex HPLC profiles without any of the desired product.

Based on the most efficient reaction time observed in Table 1, the aq. NaOH/toluene solvent/base system with *t*-butyl ester alkylating agent was used to screen a further dozen catalysts covering a wider range of benzylic substituents (e.g., 2-CN-Bn and anthracenyl) and O-allyl derivatives. The reaction conditions were kept consistent. Unfortunately, the effects observed for the cinchona alkaloid-based catalysts could not be fully rationalised (Entries 3G to 3R in Table 2).

For instance, comparison of Entries 3Q and 3R showed that the O-allyl substituent had a strong impact upon the e.e., yet the results of a similar comparison between Entries 1B and 3P showed no significant effect. Elucidating the effect of the substituent on the quinoline ring was similarly difficult, e.g., comparing Entries 1A and 1B (~no difference) or Entries 3I with 3L (large difference). However, there seemed to be a definite trend in terms of the steric bulk of the -CH_2_Ar group at this stage. In particular, Entries 3K and 3R, where the very largest groups were used, showed promising enantioselectivity.

**Scheme 5:**
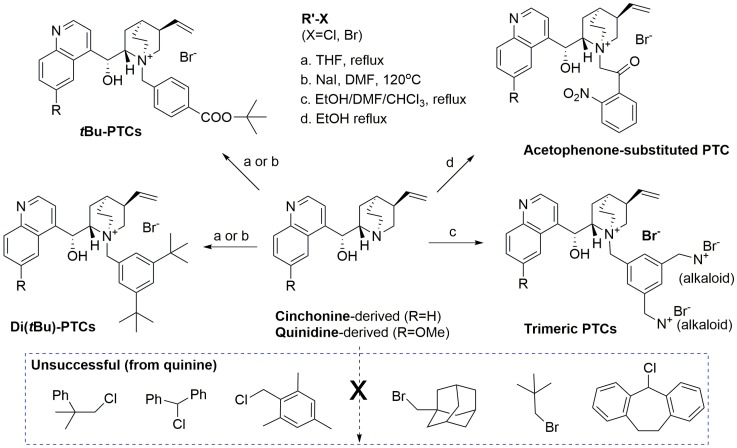
Successful and unsuccessful attempts of bulky PTCs preparation.

As a result, preparation of super-bulky PTCs was planned for further investigation (Scheme 5). The work commenced with quinine, followed by cinchonidine. Disappointingly, many of the alkylating agents bearing α-branched or neopentylic groups were very unreactive under both the standard conditions employed [refluxing tetrahydrofuran (THF)) and under more forcing conditions[catalytic sodium iodide, dimethylformamide (DMF), 120 °C, over 48 h]. A few successful reactions were conducted to afford a small set of bulkier PTCs including two literature PTCs, a trimeric PTC made using 1,3,5-tris-(bromomethyl)benzene [32] and acetophenone-substituted PTCs made using 2-bromo-2′-nitroacetophenone [33]. These latter PTCs may form a stabilised nitrogen ylide as the active base, given the acidity of the protons of the acetophenone substituent. Whether this would have any effect, however, was unclear.

In parallel, since the catalysts were typically formed from the alkaloid and a benzylic halide derivative, several novel catalysts were made from the reaction mixture of TBBMB alkylating agent and the parent alkaloid. All new catalysts were tested (Entries 4 in Table 2), however, none of these catalysts performed better than the existing ones, although Entries 4U and 4Z were comparable. Overall, few conclusions can be drawn from these results. Interestingly, the catalyst formed in situ using cinchonidine proved moderately selective (Entry 4Z), despite the absence of a bulky substituent in the 2- or 3-position of the aromatic ring. While not quite as selective as others, the ease of use (adding only a cheap alkaloid to the reaction mixture) was certainly attractive.

Chiral PTC-promoted alkylations using the analogous (Me)-ester alkylating agent, such as MBMB, were subsequently investigated. The results in Entries 2D and 2K using weaker carbonate bases suggested (Me)-ester alkylating agent might be compatible with these milder PTC conditions. A small set of reactions using *N*-(2-NO_2_-Bn)-quinidinium bromide (K_2_CO_3_ as base) were explored. In parallel, the benzylic chloride electrophile was also tested. MBMB was sufficiently stable under these reaction conditions to afford the desired (Me)-ester **4**. However, as expected, the reaction was slow even at 60 °C and after heating for 3 h it reached only ~35% (area) conversion. The measured e.e. after extended heating (~14 h, 60 °C) was 18% of major enantiomer with *R* configuration (compared with 30% for the *t*-Bu analogue under the same conditions). Meanwhile, under the same conditions, the reaction with analogous methyl(4-chloromethyl)benzoate was too slow to be explored further, giving only a trace of product. Prolonged heating resulted in the generation of significant impurities. Two reactions, 5K & 5R in Table 2, were set up on 5 mmol scale using the same catalysts as in Entries 3K and 3R, but with (Me)-ester alkylating agent instead. Despite both reactions being slow, they eventually reached >95% conversion of ketone **3** after ~2 days, with Entry 5R completing first. The e.e. measured was 38% after purification, with the later-eluting (*S*)-enantiomer being the major, as confirmed by chiral HPLC analysis against (*S*)-**4** reference standard. A portion of (*rac*)-**4** was carried through the diastereoselective reduction using TiBA with desired (*S*)-diastereoisomer (87%) being predominant. Subsequent hydrolysis afforded a mixture consisting primarily of desired diastereoisomer [(*S*,*S*)-**7** at 6.2 min & (*R*,*R*)-**7** at 5.9 min] as confirmed by chiral HPLC against the reference standards. This therefore demonstrated that the choice of PTC should be cinchonidine or quinine derived.

Thus far, the catalysts with a free -OH group or substituted with an allyl group have been described. However, it was quite possible that the -OH group of unsubstituted PTCs could be functionalised during the reaction by the reactive alkylating agent used. This could lead to either a more selective or a less selective catalyst, or perhaps both could operate in tandem. At least one literature study has demonstrated facile *O*-alkylation of a cinchona alkaloid-derived PTC under similar conditions [34]. Despite this, most authors continue to describe the catalyst as the -OH form. Indeed a recent paper [35] included a mechanistic explanation based upon H-bonding with this group, although this explanation seemed to go against the general consensus that it was the cationic N^+^ that interacts with the enolate [36].

### 2.3. PTC-Promoted Alkylation of Unsaturated Ketone ***9***

**Scheme 6:**
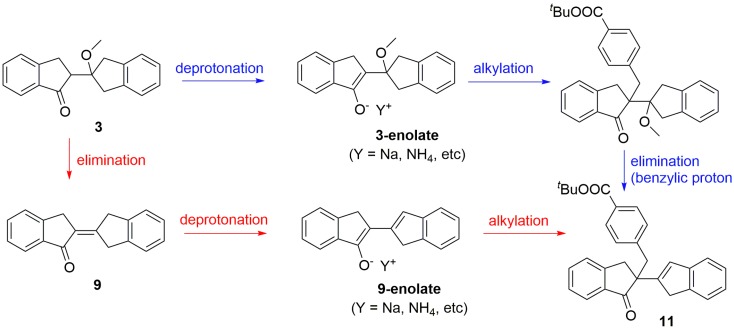
Two possible routes for PTC promoted alkylation/elimination of ketone **3**.

Chiral analysis of the achiral PTC reaction of ketone **3** with TBBMB proved complex due to the presence of impurities eluting close to the peaks of interest (this was not a problem when analysing the analogous reactions using MBMB, whose products eluted later on the chiral column). It was thought the formation of the unsaturated ketone **9** under basic conditions could account for one of the side-products observed. As a result, it was decided to expand the investigation to the ketone **9** and PTC alkylation of such ketone. Unsaturated ketone **9** was made in moderate yield 73% according to the published method [37], using ketone **3** in a mixture of methanol (MeOH)/DCM with catalytic trifluoromethanesulfonic acid under reflux conditions. The synthesis of ketone **9** had a further, perhaps more important driver. Previous reactions gave only suggestions as to whether the enolate that reacts with the alkylating agent in PTC reactions was derived from ketone **3**, or if unsaturated ketone **9** was formed in situ and was then deprotonated (Scheme 6).

In fact, either or both routes were possible and it would be challenging to distinguish between these unless intermediates could be isolated. Indeed, the degree to which either route operates could also depend heavily upon catalyst structure, which could be one reason why it was difficult to identify clear trends in selectivity as a function of catalyst structure. Since unsaturated ketone **9** would form an extended and near-planar, highly conjugated enolate while ketone **3** would not, substrate-catalyst interactions could be quite different for each of the two pathways. Alkylation of ketone **9** was tested using TBBMB. The HPLC profiles showed similar results to those of ketone **3**, but with slightly fewer impurities (Table 3). Most importantly, a clear improvement was shown with at least 3 out of 4 catalysts when compared to the reactions of ketone **3** under the same conditions.

In each case, HPLC analysis was performed after 3 h and 21 h showing very little reaction progress after the initial 3 h. The level of both starting materials in Entries 6K, 6R and 6Z were <10% (by area), but the level in Entry 6D was higher (15–20% by area); dissolution of unsaturated ketone **10** also took noticeably longer (>2 h) in this reaction. This suggested that the reaction stalled before completion, possibly due to catalyst decomposition, derivatisation or a change in solubility profile. As with other reactions, precipitation of solids was observed, but these were not further identified.

In the original control reaction with ketone **3** alone under achiral phase transfer conditions, much of the starting material remained following overnight reaction, while the achiral PTC alkylation of ketone **3** was rapid [10]. This demonstrated that the conversion from ketone **3** to unsaturated ketone **9** under the reaction conditions was not facile, or that the elimination was readily reversible. The results suggested that alkylation takes place via **3**-enolate or through both pathways. In contrast, only a single pathway is possible with unsaturated ketone **9**. These results suggested that higher selectivity might be possible with this new substrate, while the absence of any ambiguity regarding the reaction pathway could make optimisation more straightforward. The substrate may also be more reactive (it should have a lower pKa than ketone **3** due to the extended conjugation, which could be important if deprotonation is rate-limiting), giving a wider scope for variation of base and temperature for future screening. In addition, it is likely that slight modifications to the synthetic method used to prepare ketone **3** could be made to afford this unsaturated ketone **9**. Given the lower solubility of **9** compared to **3**, product isolation might even be made easier, or isolated yields higher.

### 2.4. Hydrolase Screening

Enzyme catalysed kinetic bio-resolution is largely used in organic and medicinal chemistry, especially in the highly controlled enantiomeric synthesis of chiral carboxylic acids [38,39,40,41,42]. Due to the chirality of the active site of the enzyme, one enantiomer fits better than its counterpart and therefore converts at a higher rate. As a result, a kinetic resolution of the racemate is achieved [43,44]. For high selectivity, the large difference in the reaction rates of the individual enantiomers should be achieved [45]. However, in many cases, the resolution does not show such differences in rates. In 1982, C. J. Sih [46] introduced a useful treatment of the kinetics of enzymatic resolutions, describing the dependency of the enantiomeric excess of substrate (eeS) and product (eeP) and the reaction conversion based on a theoretical basis laid by Sharpless [47] and Fajans [48]. Enantiomeric ratio (E), a selectivity parameter of a resolution, was introduced, which remains constant throughout the reaction and is only determined by the environment of the system. This method was further developed in the 1990s [49,50]. The relationship between the selectivity of a reaction (E value) and the optical purity of both substrate (eeS) and product (eeP) was expressed in the following equation:

$$E=\frac{\ln\frac{\left[\mathrm{eeP}\left(1-\mathrm{eeS}\right)\right]}{\left(\mathrm{eeP}+\mathrm{eeS}\right)}}{\ln\frac{\left[\mathrm{eeP}\left(1+\mathrm{eeS}\right)\right]}{\left(\mathrm{eeP}+\mathrm{eeS}\right)}}$$

The expected optical purity of a substrate can be calculated for a chosen point of conversion and the E value can be determined as a convenient constant value for the “selectivity” of the resolution. It has well been accepted that E-values of <8 is not a useful resolution; ~8–30 is regarded to yield e.e. from moderate to good; >30–100 is regarded to yield e.e. from good to excellent; >100 is regarded to yield excellent e.e. of both enantiomers [51].

(*Rac*)-**4** was screened against a panel of 48 commercial hydrolase enzymes in the presence of two different organic cosolvents, since organic cosolvents strongly influence the activity and/or selectivity of many enzymes for a given substrate. Hydrolase screening using DMSO as cosolvent resulted in four positive hits. AH-06 and AH-24 exhibited only trace levels of conversion and no discernible e.e. was observed in either case. AH-09 and AH-46 however showed some selectivity for the desired transformation (Table 4).

No positive hits were observed for the screen using 2-MeTHF as cosolvent. In an effort to improve upon the modest enantio-selectivity observed with DMSO, a further extensive solvent screen was carried out on (*rac*)-4 with the most promising enzymes: AH-09, AH-24 and AH-46. Eighteen cosolvent systems were investigated in total, including hexane, pentane, toluene, DMSO, DMF, diethyl ether, THF, dioxane, 2-MeTHF, methyl *tert*-butyl ether (MtBE), DCM, chloroform, ethyl acetate, acetonitrile, ethanol, Isopropanol (IPA), butanol and a reaction with no organic cosolvent present. Some representative results of these screens after 72 h reaction time are outlined in Table 5.

**Figure 1:**
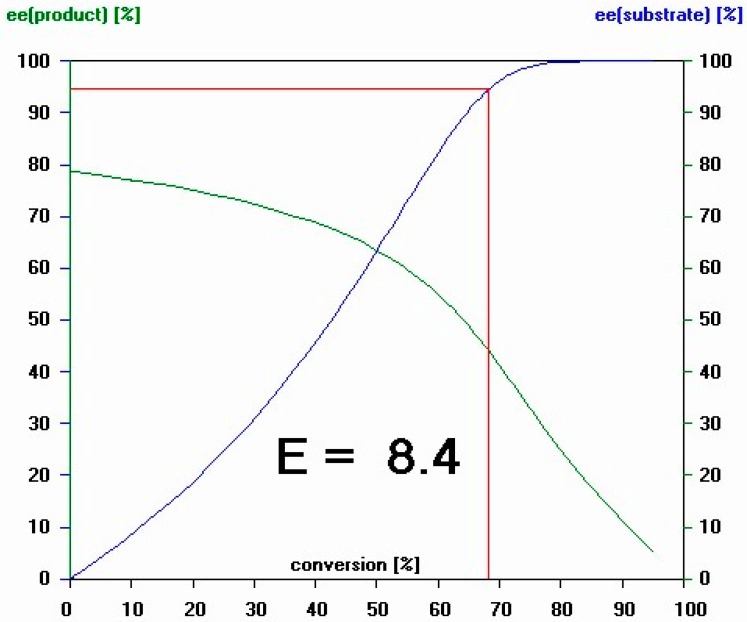
Plot of substrate & product e.e. vs. conversion for kinetic resolution of (*rac*)-**4**.

Significant improvement was achieved in Entries 18 and 19, where MtBE as cosolvent gave the best result with a selectivity factor of 8.4 (Figure 1). As AH-46 hydrolyses (*R*)-**4** preferentially over its enantiomer (Table 5), it is possible to isolate (*S*)-**4** with an e.e. of 95% in approximately 35% yield by driving the reaction to 68% conversion as shown in Scheme 2. It was believed that further optimisation screening reaction could be performed on the bio-resolution reaction of (*rac*)-**4** using AH-46 with MtBE as cosolvent to further polish the E value, which includes a number of parameters, namely, temperature, concentration, pH, % cosolvent loading, % enzyme loading, % substrate loading, salt additives and the organic cosolvent:aqueous buffer ratio. These investigations are currently ongoing.

## 3. Materials and Methods

### 3.1. General Information

Chemical reagents, enzymes and solvents were obtained from commercial suppliers and used without further purification. The hydrolase enzymes were from an Almac screening kit (Almac, Craigavon, UK). (*rac*)-**4**, enantiomeric compounds [(*S*,*S*)-**7**, (*R*,*R*)-**7**, (*R*,*S*)-**8**, (*S*,*R*)-**8**] and diastereoisomers **5** and **6**, were obtained internally within our group.

Proton Nuclear Magnetic Resonance (NMR) spectra were recorded at 27 °C on a DPX 400 MHz spectrometer (Bruker, Coventry, UK) using solvents CDCl_3_ and referenced relative to residual CDCl_3_ (δ = 7.26 ppm). Chemical shifts are reported in ppm and coupling constants (*J*) in Hertz. Carbon NMR spectra were recorded on the same instruments (100 MHz) with total proton decoupling. NMR spectra were analysed with Bruker TopSpin 3.5 NMR software (Bruker, Coventry, UK). ESI mass spectra were acquired using a Micromass LCT-time of flight mass spectrometer (TOF) interfaced to a Waters 2690 HPLC (Waters, Hertfordshire, UK). The instrument was operated in positive or negative mode as required. EI mass spectra were acquired using a GCT Premier Micromass TOF instrument (Waters, Hertfordshire, UK). The instrument was operated in positive mode. Chemical Ionisation (CI) mass spectra were determined using a GCT Premier Micromass mass spectrometer (Waters, Hertfordshire, UK) in CI mode utilizing methane as the ionisation gas. Flash chromatography was carried out using silica gel, particle size 0.04–0.063 mm. TLC analysis was performed on precoated 60F_254_ slides, and visualised by UV irradiation. Specific optical rotation was measured at 22 °C in CHCl_3_.

Chiral HPLC analyses were conducted on a Thermo Separations Products LC (ThermoFisher, Paisley, UK) equipped with a binary pump, column oven and variable wavelength detector with data processing using ChromQuest software (v5.0, ThermoFisher, Paisley, UK). Solvents (*n*-heptane, ethanol and MeOH) and additive trifluoroacetic acid (TFA) were all of HPLC solvent grade. Samples were prepared at 0.5 mg/mL in the eluent mixture. Chromatographic conditions for PTC reactions: Daicel Chiralpak IC column (250 × 4.6 mm, 5 µm) was used with isocratic elution with mobile phase *n*-heptane:IPA:TFA. 90:10:0.1 (*v*/*v*/*v*) for individual enantiomers of compounds **7** & **8** with 40 min of run-time; 95:5:0.1 (*v*/*v*/*v*) for (*R*)-**10** & (*S*)-**10** with 40 min of run-time; 86:14:0.1 (*v*/*v*/*v*) for compounds **3** & **11** with 20 min of run-time; flow rate was 1.0 mL/min; column oven & auto-sampler temperatures are ambient; injection volume was10 µL; injection loop size was 100 µL and detector wavelength was 254 nm. The chiral HPLC chromatograms of (*R*,*R*)-**7** (RT = 6.1 min), (*S*,*S*)-**7** (RT = 6.5 min), (*R*,*S*)-**8** (RT = 10.0 min), (*S*,*R*)-**8** (RT = 12.2 min), (*R*)-**4** (RT = 18.5 min), (*S*)-**4** (RT = 35.8 min), (*R*)-**10** (RT = 22.0 min), (*S*)-**10** (RT = 29.1 min), (*E1*)-**3** (RT = 8.3 min), (*E2*)-**3** (RT = 8.5 min), (*E1*)-**11** (RT = 7.7 min), (*E2*)-**11** (RT = 11.6 min), are shown in Figures S7–S11 in Supplementary Materials.

A separate chiral HPLC method was further developed for hydrolase screening to provide better separation of (*rac*)-**4** and the enantiomers of (*S*)-**10** and (*R*)-**10** with shorter retention time. Daicel Chiralpak AD-H column (250 × 4.6 mm, 5 µm) was used. The flow rate was 1.2 mL/min with isocratic elution with mobile phase: *n*-heptane:IPA:TFA 80:20:0.1 (*v*/*v*/*v*) and 20 min of run time. The detector wavelength was 254 nm and column temperature was ambient. The chromatogram was given in Figure S12 in the Supplementary Materials, showing RT = 10.4 min for (*R*)-**4**, RT = 11.6 min for (*S*)-**4**, RT = 15.5 min for (*R*)-**10** and RT = 17.6 min for (*S*)-**10**.

### 3.2. General Producre for the Sysnthesis of Chiral Standards: *(*S*)-**4*** and *(*R*)-**4***

The hydroxycarboxylic acid **7** or **8** (1 eq.) was taken up in a mixture of toluene and MeOH (3:2, *v*/*v*) and stirred vigorously. Trimethylsilyldiazomethane (2.0 M in diethyl ether, 2.3 eq.) was added dropwise from a syringe until the characteristic yellow colour persisted. TLC analysis was used to confirm complete conversion of the acid. The solvent was removed under reduced pressure yielding the intermediate methyl ester. The crude hydroxyl ester was used without further purification. Chromium trioxide (6.0 eq.) was added to a solution of dry pyridine (2.0 eq.) in dry DCM. The resulting mixture was stirred at room temperature for 15 min and a solution of the crude hydroxyl ester (1.0 eq.) in dry DCM was added rapidly. After 15 min the mixture was decanted and the remaining solid was extracted with diethyl ether. The combined organic extracts were washed with 5% aq. NaOH, 5% aq. HCl, 5% aq. NaHCO_3_ and brine. The organic layer was dried over sodium sulphate and concentrated under reduced pressure yielding the target keto ester (*S*)-**4** or (*R*)-**4**. The details of each reaction procedure and ^1^H-NMR spectra of each product are given in the Supplementary Materials (Figures S1–S4).

#### 3.2.1. Methyl (*S*)-4-((1′-oxo-1′,3′-dihydro-1*H*,2′*H*-[2,2′-biinden]-2′-yl)methyl)benzoate (*S*)-**4**

Enantiomeric acid (*S*,*S*)-**7** (0.20 g) was used to give (*S*)-**4** (0.14 g, 67%) as pale yellow solid. [α]_D_ +168.9° (1 mg in 1 mL of CHCl_3_); ^1^H-NMR (400 MHz, CDCl_3_) δ_H_ 3.32–3.61 (m, 6H, 3 × CH_2_), 3.89 (s, 3H, CH_3_), 6.74 (s, 1H, C=CH), 7.15–7.30 (m, 5H, Ar-H), 7.36 (t, *J* = 7.68 Hz, 2H, Ar-H), 7.40 (d, *J* = 7.17 Hz, 2H, Ar-H), 7.57 (t, *J* = 7.68 Hz, 1H, Ar-H), 7.75 (d, *J* = 8.19 Hz, 1H, Ar-H), 7.83 (d, *J* = 8.71 Hz, 2H, Ar-H); ^13^C-NMR δ_C_ (100 MHz, CDCl_3_): 37.2 (CH_2_), 38.4 (CH_2_), 41.8 (CH_2_), 51.6 (CH_3_), 56.7 (quat. C), 120.3 (tert. C), 123.1 (tert. C), 124.2 (tert. C), 124.3 (tert. C), 125.7 (tert. C), 126.0 (tert. C), 127.3 (tert. C), 128.0 (quat. C), 128.2 (tert. C), 2 × 129.0 (2 × tert. C), 2 × 129.6 (2 × tert. C), 134.7 (tert. C), 2 × 142.5 (quat. C), 142.7 (quat. C), 143.7 (quat. C), 148.3 (quat. C), 151.8 (quat. C), 166.5 (COOCH_3_), 205.2 (C=O). HRMS (+H^+^): 395.1657 *m*/*z*; required 395.1642; C_27_H_23_O_3_. Chiral HPLC: Chiralpak IC, heptane:IPA:TFA (90:10:0.1, *v*/*v*/*v*), RT 35.8 min, 98.7% e.e.

#### 3.2.2. Methyl (*R*)-4-((1′-oxo-1′,3′-dihydro-1*H*,2′*H*-[2,2′-biinden]-2′-yl)methyl)benzoate (*R*)-**4**

Enantiomeric acid (*R*/*R*)-**7** (0.20 g) was used to give (*R*)-**4** (0.16 g, 76%) as pale yellow solid. [α]_D_ −167.9° (1 mg in 1 mL of CHCl_3_); ^1^H-NMR (400 MHz, CDCl_3_) δ_H_ 3.32–3.61 (m, 6H, 3 × CH_2_), 3.89 (s, 3H, CH_3_), 6.74 (s, 1H, C=CH), 7.16–7.30 (m, 5H, Ar-H), 7.36 (t, *J* = 7.43 Hz, 1H, Ar-H), 7.41 (t, *J* = 7.82 Hz, 1H, Ar-H), 7.57 (t, *J* = 7.43 Hz, 1H, Ar-H), 7.75 (d, *J* = 7.82 Hz, 1H, Ar-H), 7.86 (d, *J* = 8.60 Hz, 2H, Ar-H); ^13^C-NMR (100 MHz, CDCl_3_) δ_C_: 37.2 (CH_2_), 38.4 (CH_2_), 41.8 (CH_2_), 51.6 (CH_3_), 56.7 (quat. C), 120.3 (tert. C), 123.1 (tert. C), 124.2 (tert. C), 124.3 (tert. C), 125.7 (tert. C), 126.0 (tert. C), 127.3 (tert. C), 128.0 (quat. C), 128.2 (tert. C), 2 × 129.0 (2 × tert. C), 2 × 129.6 (2 × tert. C), 134.7 (tert. C), 2 × 142.5 (quat. C), 142.7 (quat. C), 143.7 (quat. C), 148.3 (quat. C), 151.8 (quat. C), 166.5 (COOCH_3_), 205.2 (C=O). HRMS (+H^+^): 395.1649 *m*/*z*; required 395.1642; C_27_H_23_O_3_. Chiral HPLC: Chiralpak IC, heptane:IPA:TFA (90:10:0.1, *v*/*v*/*v*), RT 18.5 min, 98.7% e.e.

### 3.3. General Producre of Hydrolysis of Chiral Standards: *(*S*)-**10*** and *(*R*)-**10***

Aqueous 30% NaOH (8.0 eq.) was added to a stirred solution of the keto (Me)-ester (*S*)-**4** or (*R*)-**4** (1.0 eq.) in MeOH. The resulting mixture was heated at 60 °C for 3 h. Water was added and the mixture was washed with DCM. The aqueous layer was acidified with 10% aq. HCl and extracted with DCM. The combined organic extracts were washed with brine and dried under reduced pressure yielding crude keto-acid. Purification by flash chromatography using 1:1 ethyl acetate:cyclohexane as eluent gave pure corresponding the keto acid (*S*)-**10** or (*R*)-**10**. The details of each reaction procedure and ^1^H NMR spectra of each product are given in Supplementary Materials (Figures S5 and S6).

#### 3.3.1. (*S*)-4-((1′-Axo-1′,3′-dihydro-1*H*,2′*H*-[2,2′-biinden]-2′-yl)methyl)benzoic acid (*S*)-**10**

Methyl ester (*S*)-**4** (0.25 g) was used to give (*S*)-**10** (0.17 g, 71%) as pale yellow solid. [α]_D_ +142.5° in CHCl_3_; ^1^H-NMR (400 MHz, CDCl_3_) δ_H_ 3.32–3.62 (m, 6H, 3 × CH_2_), 6.74 (s, 1H, C=CH), 7.15–7.30 (m, 5H, Ar-H), 7.36 (t, *J* = 7.24 Hz, 1H, Ar-H), 7.41 (d, *J* = 8.30 Hz, 2H, Ar-H), 7.57 (t, *J* = 7.49 Hz, 1H, Ar-H), 7.75 (d, *J* = 7.76 Hz, 1H, Ar-H), 7.93 (d, *J* = 8.12 Hz, 1H, Ar-H); ^13^C-NMR (100 MHz, CDCl_3_) δ_C_ 37.2 (CH_2_), 38.4 (CH_2_), 41.8 (CH_2_), 56.7 (quat. C), 120.3 (tert. C), 123.1 (tert. C), 124.2 (tert. C), 124.4 (tert. C), 125.7 (tert. C), 126.0 (tert. C), 127.1 (quat. C), 127.3 (tert. C), 128.3 (tert. C), 2 × 129.6 (2 × tert. C), 2 × 129.7 (2 × tert. C), 134.7 (quat. C), 134.8 (tert. C), 142.6 (quat. C), 143.5 (quat. C), 143.6 (quat. C), 148.2 (quat. C), 151.8 (quat. C), 171.3 (COOH), 205.2 (C=O). HRMS (+Na^+^): 403.1305 *m*/*z*; required 403.1304; C_26_H_20_O_3_Na. Chiral HPLC: Chiralpak IC, heptane:IPA:TFA (95:5:0.1, *v*/*v*/*v*), RT 29.1 min, 98.8% e.e.

#### 3.3.2. (*R*)-4-((1′-Oxo-1′,3′-dihydro-1*H*,2′*H*-[2,2′-biinden]-2′-yl)methyl)benzoic acid (*R*)-**10**

(Me)-ester (*R*)-**4** (0.25 g) was used to give (*R*)-**10** (0.15 g, 63%) as white solid. [α]_D_ −121.3° in CHCl_3_; ^1^H-NMR (400 MHz, CDCl_3_) δ_H_ 3.32–3.62 (m, 6H, 3 × CH_2_), 6.74 (s, 1H, C=CH), 7.15–7.31 (m, 5H, Ar-H), 7.36 (t, *J* = 7.24 Hz, 1H, Ar-H), 7.41 (d, *J* = 8.30 Hz, 2H, Ar-H), 7.57 (t, *J* = 7.49 Hz, 1H, Ar-H), 7.75 (d, *J* = 7.76 Hz, 1H, Ar-H), 7.93 (d, *J* = 8.12 Hz, 1H, Ar-H); ^13^C-NMR (100 MHz, CDCl_3_) δ_C_ 37.2 (CH_2_), 38.4 (CH_2_), 41.8 (CH_2_), 56.7 (quat. C), 120.3 (tert. C), 123.1 (tert. C), 124.2 (tert. C), 124.4 (tert. C), 125.7 (tert. C), 126.0 (tert. C), 127.1 (quat. C), 127.3 (tert. C), 128.3 (tert. C), 2 × 129.6 (2 × tert. C), 2 × 129.7 (2 × tert. C), 134.7 (quat. C), 134.8 (tert. C), 142.6 (quat. C), 143.5 (quat. C), 143.6 (quat. C), 148.2 (quat. C), 151.8 (quat. C), 171.3 (COOH), 205.2 (C=O). HRMS (-H^+^): 379.1329 *m*/*z*; required 379.1336; C_26_H_19_O_3_. Chiral HPLC: Chiralpak IC, heptane:IPA:TFA (95:5:0.1, *v*/*v*/*v*), RT 22.0 min, 95.6% e.e.

### 3.4. General Procedure of Synthesis of PTCs (Cinchona Alkaloids Derived)

The alkaloid (12.3 mmol, 1 eq.) and the appropriate substituted benzylic halide derivative (12.3 mmol, 1 eq.) were dissolved in THF (40 mL) with addition of a trace of NaI. The mixture was heated to reflux overnight and then cooled and stirred at ambient temperature for 1 h. In most cases the product precipitated as an off-white solid, but where this was not the case and the mixture contained only a small amount of solid or no solid at all, then diethyl ether (20 mL) was added dropwise. The solid was removed via filtration and washed with THF (50 mL) or ether:THF, (1:1, *v*/*v*, 50 mL) and was dried under reduced pressure at 40 °C. Where the solid formed was not a fine powder it was then taken up in DCM and this solution was then added dropwise to rapidly stirring ether (100 mL). This usually gives a finely divided solid that could be filtered and dried. (Note: The cinchonine derived PTCs are usually very insoluble. The quinidine derived PTCs are often completely soluble at the end of the reaction.) The di(*t*-butyl)benzyl PTC was prepared according to the standard procedure above and was filtered directly from the reaction mixture.

### 3.5. Representative Procedure for PTC Alkylations Using MBMB (Entry 5R)

The reaction was carried out in a 100 mL Quickfit Erlenmeyer flask heated in an oil bath. To a stirred solution of ketone **3** (1.39 g, 5.0 mmol), PTC (40 mg/mmol) and MBMB (1.15 g, 5.0 mmol) in toluene (50 mL) was added 25% aq. K_2_CO_3_ (10 mL) at 35 °C. The reaction mixture was stirred in the heating bath overnight; HPLC analysis of toluene phase indicated ~40% e.e., but conversion was poor. The reaction temperature was then increased to 70 °C. After stirring for 7 h at this temperature (and over a weekend at RT, though it is unlikely that any significant conversion took place during this time), the peak of ketone **3** was <5% by area. The mixture was diluted with toluene and water, and the layers separated. The toluene portion was washed twice with dilute aqueous HCl (~1 M), once with brine, dried over MgSO_4_, filtered and concentrated under reduced pressure to afford the crude product. The product was chromatographed using the dry-flash technique on silica with toluene as eluent to afford (Me)-ester **4** (0.83 g, 38% e.e., 41%) as an oil that solidified on standing. Some further product was contained in mixed fractions that were not further purified. ^1^H-NMR (400 MHz, DMSO-*d*_6_) δ_H_ 7.72–7.68 (m, 1H), 7.64 (d, *J* = 7.4 Hz, 1H), 7.53 (d, *J* = 7.6 Hz, 1H), 7.39 (t, *J* = 7.2 Hz, 1H), 7.26 (t, *J* = 8.0 Hz, 2H), 7.18 (t, *J* = 7.4 Hz, 1H), 7.10 (td, *J* = 7.4, 1.1 Hz, 1H), 6.74 (s, 1H), 3.56–3.32 (m, 4H; 2 × AB system), 1.49 (s, 5H).

### 3.6. Hydrolase Screening Conditions

The enzymes employed in the screening are described in Table 6. The general screening conditions are outlined below. Each reaction mixture contained the following: 3 mg of (*rac*)-**4**, 1 mL of KH_2_PO_4_ buffer solution (0.1 M, pH = 7), 50 µL of DMSO (cosolvent system 1) or 200 µL of 2-MeTHF (cosolvent system 2), 5–10 mg of hydrolase enzyme. The reaction mixture was then shaken for 18 h at 30 °C. After stirring overnight, 1 mL of 2-MeTHF was added to each reaction mixture. 150 µL of 10% H_3_PO_4_ was used to adjust the pH to ~2. The reaction vessels were shaken for 5 min which resulted in the formation of emulsions in some cases. Centrifugation of the biphasic reaction media might be used to achieve adequate phase separation. TLC analysis was performed using hexane:ethyl actetate (7:3, *v*/*v*) as eluent. 60 µL of aliquots were taken from the organic phase of positive TLC hits and filtered through MgSO_4_, which was further washed with 1 mL of 2-MeTHF. The combined organic solvent was evaporated and the residue was redissolved in the required HPLC solvent mixture and analysed by chiral HPLC using enantiomers (*S*)-**10** and (*R*)-**10** as reference standards.

## 4. Conclusions

Based on the recent method development of diastereoselection in the reduction of ketone **4**, the current study aimed to increase the overall yield of PH46A synthesis on multi-kilo scale further by developing an enantioselective method. The first phase of the work was the investigation of chiral PTC promoted alkylation of ketone **3**. Twenty-six chiral PTCs, mostly derived from cinchona alkaloids, were tested. An e.e. up to 46% were achieved and it was identified that the required enantiomer for the synthesis of PH46A with (*S*,*S*) configuration was produced by catalysts from quinine or cinchonidine. Promising e.e. of up to 50% were also achieved even at this early stage using unsaturated ketone **9** as an alternative substrate, which would appear to be the better substrate for further optimisation work.

In the second phase, 48 hydrolase enzymes were screened against (*rac*)-**4** in the presence of two cosolvent systems (DMSO and 2-MeTHF). These screens identified two lead hydrolase enzymes (AH-09 and AH-46) which were further evaluated by extensive cosolvent screening (18 cosolvent systems) against (*rac*)-**4**. Positive results were obtained yielding suitable reaction conditions of enzyme AH-46 with MtBE cosolvent providing an observed selectivity factor of 8.4 with an e.e. of 77% for (*R*)-**10**, which could afford unreacted and desired (*S*)-**4** in isolation with higher e.e. up to 95%. Studies to enhance the enantioselectivity of both chiral PTC alkylation and enzymatic hydrolysis approaches are currently ongoing. If successful, we believe that the enantioselective method will increase the overall yield of PH46A and could reduce overall manufacturing cost up to 25%, especially the costly simulated moving bed chromatography purification step will no longer be required.

## Figures and Tables

**Table 1:** Chiral PTC promoted alkylation of ketone **3** and *t*-butyl(4-bromomethyl)benzoate.

Entry	PTC & Conditions	Time	e.e. (Major Enantiomer ^a^)
1A	*N*-Bn-quininium chloride, TOL:25% aq. NaOH (5:1)	<2 h	11% (*S*)
1B	*N*-Bn-cinchonidinium chloride, TOL:25% aq. NaOH (5:1)	<2 h	12% (*S*)
1C	*N*-(2-MeO-Bn)-quinidinium bromide, TOL:25% aq. NaOH (5:1)	<2 h	12% (*R*)
1D	*N*-(2-NO_2_-Bn)-quinidinium bromide, TOL:25% aq. NaOH (5:1)	6 < t < 20 h	24% (*R*)
1E	*N*,*N*′-Me_2_-ephedrinium bromide, TOL:25% aq. NaOH (5:1)	2 < t < 6 h	4% (*R*) ^b^
1F	*N*-Bn-*N*′-Me-ephedrinium bromide, TOL:25% aq. NaOH (5:1)	2 < t < 6 h	12% (*S*)
2A	*N*-(2-NO_2_-Bn)-quinidinium bromide, TOL/25% aq. NaOH (5:1)	<16 h RT	22% (*R*)
2B	*N*-(2-NO_2_-Bn)-quinidinium bromide, TOL/12% aq. NaOH (5:1)	<16 h RT	25% (*R*)
2C	*N*-(2-NO_2_-Bn)-quinidinium bromide, TOL/6% aq. NaOH (5:1)	<16 h RT	25% (*R*)
2D	*N*-(2-NO_2_-Bn)-quinidinium bromide, TOL/25% aq. K_2_CO_3_ (5:1)	16 h RT + <6 h 70 °C	34% (*R*) ^c^
2E	*N*-(2-NO_2_-Bn)-quinidinium bromide, TOL/6% aq. KOH (5:1)	<16 h RT	23% (*R*)
2F	*N*-(2-NO_2_-Bn)-quinidinium bromide, THF/MtBE (1:1)/25% aq. NaOH	<16 h RT	No expected product
2G	*N*-(2-NO_2_-Bn)-quinidinium bromide, DCM/25% aq. NaOH (5:1)	<16 h RT	9% (*S*)
2H	*N*-(2-NO_2_-Bn)-quinidinium bromide, THF/solid NaOH (1:50, *v*:*w*)	<16 h RT	No expected product
2I	*N*-(2-NO_2_-Bn)-quinidinium bromide, MtBE/25% aq. NaOH (5:1)	<16 h RT	14% (*R*)
2J	*N*-(2-NO_2_-Bn)-quinidinium bromide, TOL/CyH (1:1), 25% aq. NaOH	<16 h RT	19% (*R*)
2K	*N*-(2-NO_2_-Bn)-quinidinium bromide, TOL/25% aq. Cs_2_CO_3_ (5:1)	>3 h 60 °C	30% (*R*) ^d^

Conditions: 0.50 mmol each reactant & 20 mg PTC (Entries 1A–F:); 0.25 mmol each reactant & 10 mg PTC (Entries 2A–K). ^a^ *S* & *R* refer to the configuration of enantiomers eluted on the chiral IC column. ^b^ Within the likely margin of error for e.e. determination. ^c^ Ketone **3** conversion >90% by area after heating. ^d^ e.e. measured at ~30% ketone **3** conversion by area. TOL: toluene. e.e.: enantiomeric excess.

**Table 2:** PTC Reactions using a Range of Catalysts.

Entry	PTC Core	-CH_2_Ar	-OR′	X^−^	Time	e.e. (Major Enantiomer ^a^)
3G	Cinchonine	-CH_2_(2-NO_2_-Ph)	-OH	Br^−^	>16 h ^b^	32% (*R*)
3H	Cinchonine	-CH_2_(2-MeO-Ph)	-OH	Br^−^	<16 h	28% (*R*)
3I	Cinchonine	-CH_2_(2-CN-Ph)	-OH	Br^−^	<16 h	15% (*R*)
3J	Cinchonine	-CH_2_(1-Np)	-OH	Cl^−^	<16 h	27% (*R*)
3K	Cinchonine	-CH_2_(8-Me-1-Np)	-OH	Cl^−^	<16 h	42% (*R*)
3L	Quinidine	-CH_2_(2-CN-Ph)	-OH	Br^−^	<16 h	4% (*R*)
3M	Quinidine	-CH2(1-Np)	-OH	Cl^−^	<16 h	33% (*R*)
3N	Quinidine	-CH_2_(2,4-Cl_2_-Ph)	-OH	Br^−^	<16 h	7% (*R*)
3O	Quinidine	-CH_2_(2-Pyr)	-OH	Cl^−^	<16 h	18% (*S*)
3P	Cinchonidine	-CH_2_Ph	-O-allyl	Br^−^	<16 h	13% (*S*)
3Q	Cinchonidine	-CH_2_(9-anthryl)	-O-allyl	Br^−^	>16 h ^b^	7% (*S*)
3R	Cinchonidine	-CH_2_(9-anthryl)	-OH	Cl^−^	<16 h	41% (*S*)
4S	Quinine	-CH_2_(3,5-di(*t*Bu)-Ph)	-OH	Br^−^	<16 h	3% (*S*)
4T	Cinchonidine	-CH_2_(3,5-di(*t*Bu)-Ph)	-OH	Br^−^	<16 h	11% (*S*)
4U	Quinine	-CH_2_C(O)(2-NO_2_-Ph)	-OH	Br^−^	~16 h	33% (*S*)
4V	Cinchonidine	-CH_2_C(O)(2-NO_2_-Ph)	-OH	Br^−^	>>16 h ^c^	23% (*S*)
4W	Quinine	-CH_2_(3,5-alkaloid^+^-Ph) ^d^	-OH	Br^−^	~16 h	9% (*S*)
4X	Cinchonidine	-CH_2_(3,5-alkaloid^+^-Ph) ^d^	-OH	Br^−^	<16 h	12% (*S*)
4Y	Quinine	-CH_2_(4-*t*BuO_2_C-Ph) ^e^	-OH	Br^−^	>16 h	26% (*S*)
4Z	Cinchonidine	-CH_2_(4-*t*BuO_2_C-Ph) ^e^	-OH	Br^−^	<16 h	35% (*S*)
5K	Cinchonine	-CH_2_(8-Me-1-Np)	-OH	Cl^−^	>16 h	46% (*R*)
5R	Cinchonidine	-CH_2_(9-anthryl)	-OH	Br^−^	>16 h	38% (*S*)

Conditions: catalysts at 0.50 or 0.25 mmol scale, PTC (40 mg/mmol, except 14W & 14X at 100 mg/mmol), toluene (10 mL/mol), 25% aq. NaOH (2 mL/mmol) at room temperature. *t*-butyl ester alkylating agent was used in all reactions, except Entries 5K & 5R used methyl ester alkylating agent. ^a^ *R* or *S* refers to the configuration of the major enantiomer eluted on the chiral IC column. ^b^ <10% ketone **3** by area at 16 h. ^c^ Conversion <<50%. ^d^ Trimeric PTC. ^e^ Presumed species formed in situ.

**Table 3:** PTC Reactions of Unsaturated Ketone **9**.

Entry	PTC Core	-CH_2_Ar	-OR′	e.e. (Major Enantiomer ^a^)	X^−^	Reference e.e. of Reaction of Ketone 3
6D	Quinidine	-CH_2_(2-NO_2_-Ph)	-OH	28% (*R*)	Br^−^	24% (*R*), 1D
6K	Cinchonine	-CH_2_(8-Me-1-Np)	-OH	50% (*R*)	Cl^−^	42% (*R*), 3K
6R	Cinchonidine	-CH_2_(9-anthryl)	-OH	50% (*S*)	Cl^−^	41% (*S*), 3R
6Z	Cinchonidine	-CH_2_(4-*t*BuO_2_C-Ph) ^b^	-OH	43% (*S*)	Br^−^	35% (*S*), 4Z

Conditions: PTC 40 mg/mmol, selected catalysts 0.25 mmol scale, toluene 10 mL/mmol, 25% aq. NaOH 10 mL/mmol, RT, 21 h. ^a^ *R* or *S* refers to the configuration of the major enantiomer eluted on chiral IC column. ^b^ Presumed species formed in situ.

**Table 4:** Positive hits for hydrolase screen of (*rac*)-**4** using DMSO as cosolvent.

Entry	Hydrolase	e.e. (Substrate)	e.e. (Product)	Major Enantiomer	E	Conversion
7	AH-06	N/A	N/A	Racemic	N/A	Trace
8	AH-09	3%	20%	*S*	1.5	16%
9	AH-24	N/A	N/A	*S*	N/A	Trace
10	AH-46	46%	46%	*R*	3	17%

e.e.: enantiomeric excess. E: enantiomeric ratio. *S* or *R* refers to configuration.

**Table 5:** Representative results of cosolvent screens of (*rac*)-**4** with selected enzymes AH-09, AH-24 and AH-46.

Entry	Hydrolase	Solvent	e.e. (Substrate)	e.e. (Product)	Major Product	E	Conversion
11	AH-09	MtBE	1%	2%	*S*	<1	3%
12	AH-09	Diethyl ether	2%	28%	*S*	1.8	6%
13	AH-09	Pentane	1%	8%	*S*	1.2	9%
14	AH-09	Hexane	1%	35%	*S*	2.1	5%
15	AH-09	DMSO	3%	20%	*S*	1.5	16%
16	AH-09	DMF	-	-	-	-	Trace
17	AH-09	Dioxane	-	-	-	-	Trace
18	AH-46	MtBE	9%	77%	*R*	8.4	10%
19	AH-46	Toluene	6%	73%	*R*	6.8	8%
20	AH-46	Pentane	1%	7%	*R*	1.2	9%
21	AH-46	Hexane	1%	13%	*R*	1.3	9%
22	AH-46	DMSO	9%	46%	*R*	3	17%
23	AH-46	Diethyl ether	-	-	-	-	Trace
24	AH-46	Dioxane	-	-	-	-	Trace
25	AH-24	DMSO	-	-	*S*	-	Trace
26	AH-24	Ethanol	-	-	-	-	Trace
27	AH-24	None	-	-	-	-	Trace

e.e.: enantiomeric excess. E: enantiomeric ratio. *S* or *R* refers to configuration.

**Table 6:** Hydrolase enzymes used to screen against (*rac*)-**4**.

Code	Hydrolase	Code	Hydrolase
AH-01	Lipase A from *Alcaligenes* sp.	AH-25	Lipase from *Rhizopus niveus*
AH-02	Lipase B from *Alcaligenes* sp.	AH-26	Protease from *Bacillus stearothermophilus*
AH-03	Lipase C from *Alcaligenes* sp.	AH-27	Lipase from *Aspergillus niger*
AH-04	Lipase from *Pseudomonas stutzeri*	AH-28	Lipase from *Penicillium roquefort*
AH-05	Lipase from *Pseudomonas cepacia*	AH-29	Protease from *Aspergillus niger*
AH-06	Lipase A from *Candida rugosa*	AH-30	Lipase from *Aspergillus oryzae*
AH-07	Lipase D from *Alcaligenes* sp.	AH-31	Protease from *Aspergillus melleus*
AH-08	Lipase E from *Alcaligenes* sp.	AH-32	Lipase from *Penicillium camembertii*
AH-09	Lipase B from *Candida rugosa*	AH-33	Protease C from *Bacillus subtilis*
AH-10	Lipase F from *Alcaligenes* sp.	AH-34	Protease B from *Aspergillus oryzae*
AH-11	Lipase from *fungal source*	AH-35	Lipase from *Pseudomonas fluorescens*
AH-12	Protease A from *Bacillus subtilis*	AH-36	Lipase A from *Burkholderia cepacia*
AH-13	Phytase	AH-37	Lipase B from *Burkholderia cepacia*
AH-14	Alkaline protease A	AH-38	Lipase A from *Rhizomucor miehei*
AH-15	Alkaline lipase A	AH-39	Lipase from *Candida antarctica*
AH-16	Lipase from *Bromeliaceae* sp.	AH-40	Lipase from *Thermomyces lanuginosus*
AH-17	Lipase from *Carica papaya*	AH-41	Protease A from *Bacillus* sp.
AH-18	Neutral protease A	AH-42	Lipase B from *Candida Antarctica (liq)*
AH-19	Alkaline protease B	AH-43	Lipase A from *Candida antarctica*
AH-20	Acidic protease A	AH-44	Protease B from *Bacillus* sp.
AH-21	Protease A from *Aspergillus oryzae*	AH-45	Lipase from *Thermomyces lanuginosus*
AH-22	Protease B from *Bacillus subtilis*	AH-46	Lipase C from *Rhizomucor miehei*
AH-23	Acylase from *Aspergillus* sp.	AH-47	Lipase Porcine *Pancrease Type II*
AH-24	Lipase B from *Candida rugosa*	AH-48	Ficin

## Supplementary Materials

Supplementary materials (the detailed experimental procedures for the syntheses of the chiral reference molecules and their corresponding NMR spectra and HPLC chromatograms) are available online.
