# Supplementary material for: Bioactive Indanes: Proof of Concept Study for Enantioselective Synthetic Routes to PH46A, a New Potential Anti-Inflammatory Agent
Source: Molecules. 2018 Jun 21;23(7):1503. doi: 10.3390/molecules23071503 (PMC6099954; doi:10.3390/molecules23071503)
Supplement: Supplementary file 1 [file molecules-23-01503-s001.pdf]

Bioactive Indanes: Proof of Concept Study for Enantioselective Synthetic Routes to PH46A, a New Potential Anti-Inflammatory Agent

Tao Zhang[1,†], Gaia Scalabrino[1,†], Neil Frankish[1,2], Helen Sheridan[1,2,*]

[1] Trino Therapeutics Ltd, The Tower, Trinity Technology and Enterprise Campus, Dublin 2, Ireland; zhangt@tcd.ie (T.Z.); gaiascal@gmail.com (G.S.)

[2] Novel Drug Discovery Group, School of Pharmacy and Pharmaceutical Sciences & Trinity Biomedical Sciences Institute, Trinity College, Dublin 2, Ireland; nfrnkish@tcd.ie (N.F.)

***** Correspondence: hsheridn@tcd.ie (H.S.); Tel.: +353-1-8962828; Fax: +353-1-89628210.

† These two authors contributed equally and should be considered joint first authors.

Supplementary Materials

Methyl (*S*)-4-((1'-oxo-1',3'-dihydro-1H,2'H-[2,2'-biinden]-2'-yl)methyl)benzoate (*S*)-4

[Synthesised from *(S,S)*-**7**]

(*S,S*)-**7** (0.20 g, 0.52 mmol, 1.0 eq.) was taken up in a mixture of toluene and methanol (MeOH) (3:2, 5 mL) and stirred vigorously. Trimethylsilyldiazomethane (2.0 M in diethyl ether, 0.60 mL, 1.2 mmol, 2.3 eq) was added dropwise from a syringe until the characteristic yellow colour persisted. TLC analysis was used to confirm complete conversion of (*S,S*)-**7**. The solvent was removed under reduced pressure yielding methyl ester of (*S,S*)-**5** (0.20 g) as a viscous yellow oil. The crude product was used without further purification. Chromium trioxide (CrO_3) (0.30 g, 3.0 mmol, 6.0 eq.) was added to a solution of dry pyridine (0.49 mL, 0.48 g, 6.0 mmol, 12.0 eq.) in dry dichloromethane (DCM) (10 mL). The resulting mixture was stirred at room temperature (r.t.) for 15 min and a solution of methyl ester of (*S/S*)-**5** (0.20 g, 0.3 mmol, 1.0 eq.) in dry DCM (1.0 mL) was added rapidly. After 15 min the mixture was decanted and the remaining tarry solid was extracted with diethyl ether (10 mL). The combined organic extracts were washed with 5% aqueous NaOH (3 x 10 mL), 5% aqueous HCl (2 x 10 mL), 5% aqueous $NaHCO_3$ (10 mL) and brine (10 mL). The organic layer was dried over sodium sulphate (Na_2SO_4) and concentrated *in vacuo* yielding (*S*)-**4** (0.10 g, 98.7% ee) as a pale yellow solid.

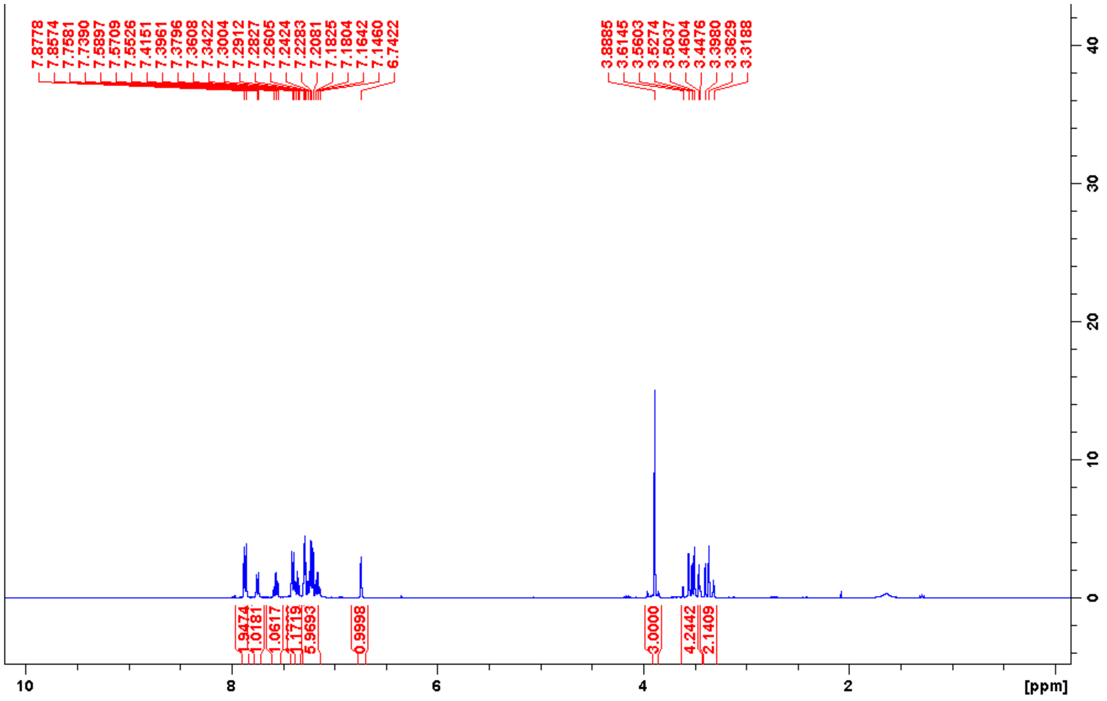

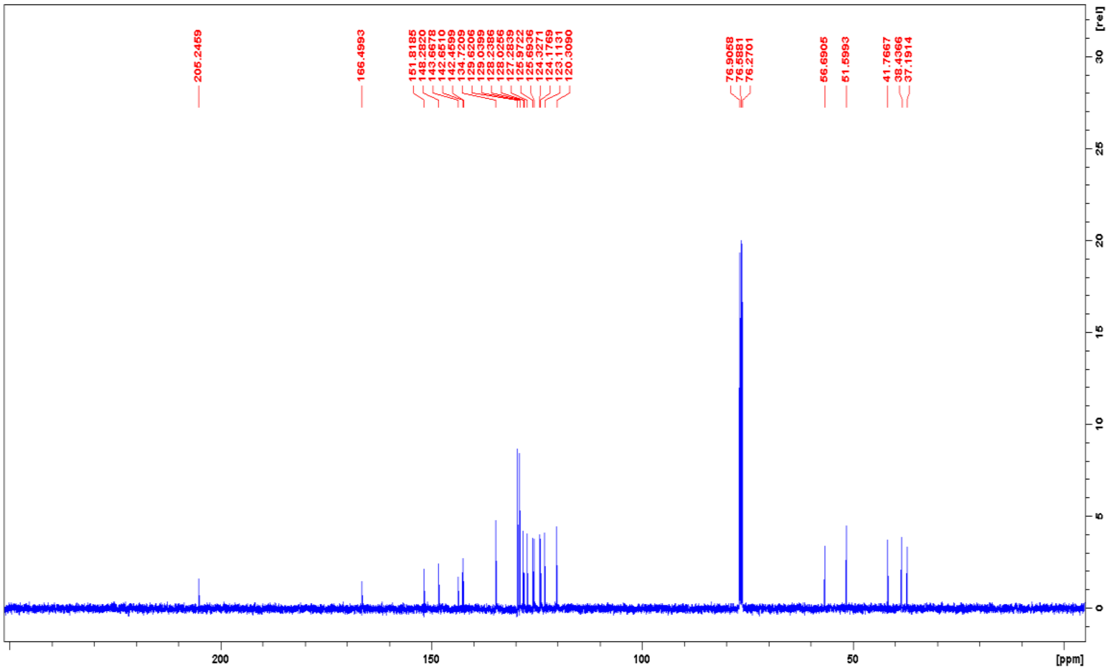

Figure S1. ^{1}H (upper) and ^{13}C (lower) NMR spectrum of (*S*)-**4** made from (*S*,*S*)-**7**.

Methyl (*R*)-4-((1'-oxo-1',3'-dihydro-1H,2'H-[2,2'-biinden]-2'-yl)methyl)benzoate (*R*)-4
[Synthesised from *(R,R)*-**7]**

(*R*,*R*)-**7** (0.20 g, 0.52 mmol, 1.0 eq.) was taken up in a mixture of toluene and MeOH (3:2, 5 mL) and stirred vigorously. Trimethylsilyldiazomethane (2.0 M in diethyl ether, 0.65 mL, 1.3 mmol, 2.5 eq) was added dropwise from a syringe until the characteristic yellow colour persisted. A TLC check was used to confirm complete conversion of (*R*,*R*)-**7**. The solvent was removed under reduced pressure yielding methyl ester of (*R*,*R*)-**5** (0.20 g) as a viscous yellow oil. The crude product was used without further purification. CrO_3 (0.30 g, 3.0 mmol, 6.0 eq.) was added to a solution of dry pyridine (0.49 mL, 0.48 g, 6.0 mmol, 12.0 eq.) in dry DCM (10 mL). The resulting mixture was stirred at r.t. for 15 min and a solution of (*R*,*R*)-**5** (0.20 g, 0.6 mmol, 1.0 eq.) in dry DCM (1.0 mL) was added rapidly. After 15 min the mixture was decanted and the remaining tarry solid was extracted with diethyl ether (10 mL). The combined organic extracts were washed with 5% aqueous NaOH (3 x 10 mL), 5% aqueous HCl (2 x 10 mL), 5% aqueous $NaHCO_3$ (10 mL) and brine (10 mL). The organic layer was dried over Na_2SO_4 and concentrated *in vacuo* yielding (*R*)-**4** (0.16 g, >93.4% ee) as a pale yellow solid.

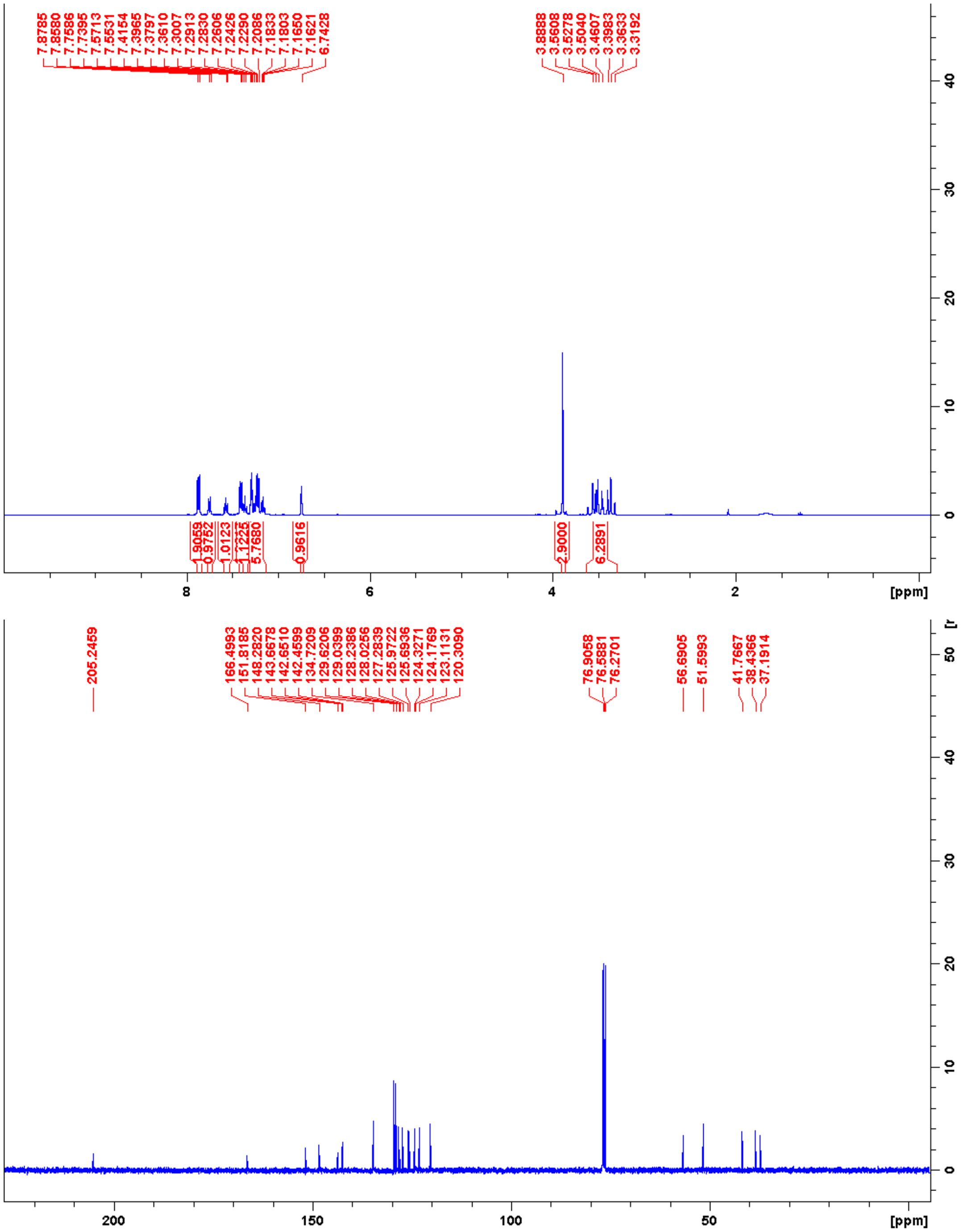

Figure S2. ^{1}H (upper) and ^{13}C (lower) NMR spectra of (*R*)-**4** synthesised from (*R*,*R*)-**7**.

Methyl (*S*)-4-((1'-oxo-1',3'-dihydro-1H,2'H-[2,2'-biinden]-2'-yl)methyl)benzoate (*S*)-4
[Synthesised from (*R*,*S*)-**8**]

(*R*,*S*)-**8** (0.10 g, 0.26 mmol, 1.0 eq.) was taken up in a mixture of toluene and MeOH (3:2, 5 mL) and stirred vigorously. Trimethylsilyldiazomethane (2.0 M in diethyl ether, 0.35 mL, 0.7 mmol, 2.7 eq) was added dropwise from a syringe until the characteristic yellow colour persisted. A TLC check was used to confirm complete conversion of (*R*,*S*)-**8**. The solvent was removed under reduced pressure yielding methyl ester of (*R*,*S*)-**6** (0.10 g) as a viscous yellow oil. The crude product was used without further purification. CrO_3 (0.15 g, 1.5 mmol, 6.0 eq.) was added to a solution of dry pyridine (0.24 mL, 0.24 g, 3.0 mmol, 12.0 eq.) in dry DCM (5 mL). The resulting mixture was stirred at r.t. for 15 min and a solution of (*R*,*S*)-**6** (0.10 g, 0.3 mmol, 1.0 eq.) in dry DCM (0.5 mL) was added rapidly. After 15 min at r.t. the mixture was decanted and the remaining tarry solid was extracted with diethyl ether (10 mL). The combined organic extracts were washed with 5% aqueous NaOH (3 x 10 mL), 5% aqueous HCl (2 x 10 mL), 5% aqueous $NaHCO_3$ (10 mL) and brine (10 mL). The organic layer was dried over Na_2SO_4 and concentrated *in vacuo* yielding (*S*)-**4** (0.10 g, >99% ee) as a pale yellow solid.

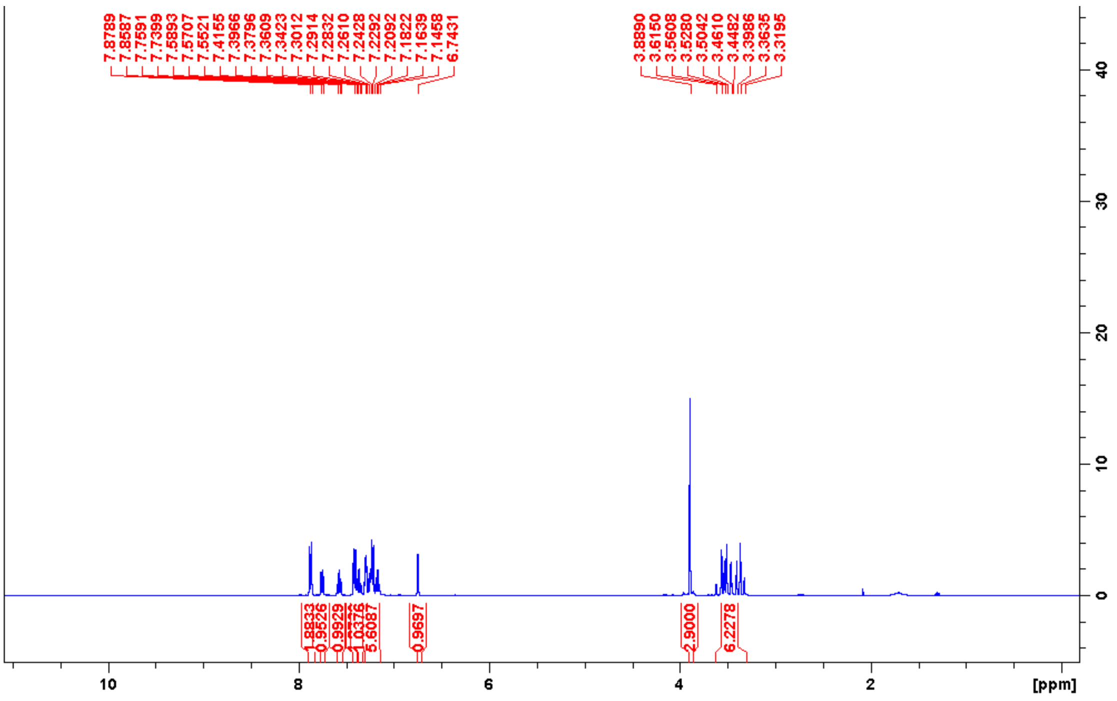

Figure S3. ^{1}H NMR spectrum of (*R*)-**4** synthesised from (*R*,*S*)-**8**.

Methyl (*R*)-4-((1'-oxo-1',3'-dihydro-1H,2'H-[2,2'-biinden]-2'-yl)methyl)benzoate (*R*)-4 (Synthesised from (*S*,*R*)-**8**)

(*S*,*R*)-**8** (0.10 g, 0.26 mmol, 1.0 eq.) was taken up in a mixture of toluene and MeOH (3:2, 5 mL) and stirred vigorously. Trimethylsilyldiazomethane (2.0 M in diethyl ether, 0.35 mL, 0.7 mmol, 2.7 eq) was added dropwise from a syringe until the characteristic yellow colour persisted. A TLC check was used to confirm complete conversion of (*S*,*R*)-**8**. The solvent was removed under reduced pressure yielding methyl ester (*S*,*R*)-**6** (0.10 g) as a viscous yellow oil. The crude product was used without further purification. CrO_3 (0.15 g, 1.5 mmol, 6.0 eq.) was added to a solution of dry pyridine (0.24 mL, 0.24 g, 3.0 mmol, 12.0 eq.) in dry DCM (5 mL). The resulting mixture was stirred at r.t. for 15 min and a solution of (*S*,*R*)-**6** (0.10 g, 0.3 mmol, 1.0 eq.) in dry DCM (0.5 mL) was added rapidly. After 15 min the mixture was decanted and the remaining tarry solid was extracted with diethyl ether (10 mL). The combined organic extracts were washed with 5% aqueous NaOH (3 x 10 mL), 5% aqueous HCl (2 x 10 mL), 5% aqueous $NaHCO_3$ (10 mL) and brine (10 mL). The organic layer was dried over Na_2SO_4 and concentrated *in vacuo* yielding (*R*)-**4** (0.08 g, >99% ee) as a pale yellow solid.

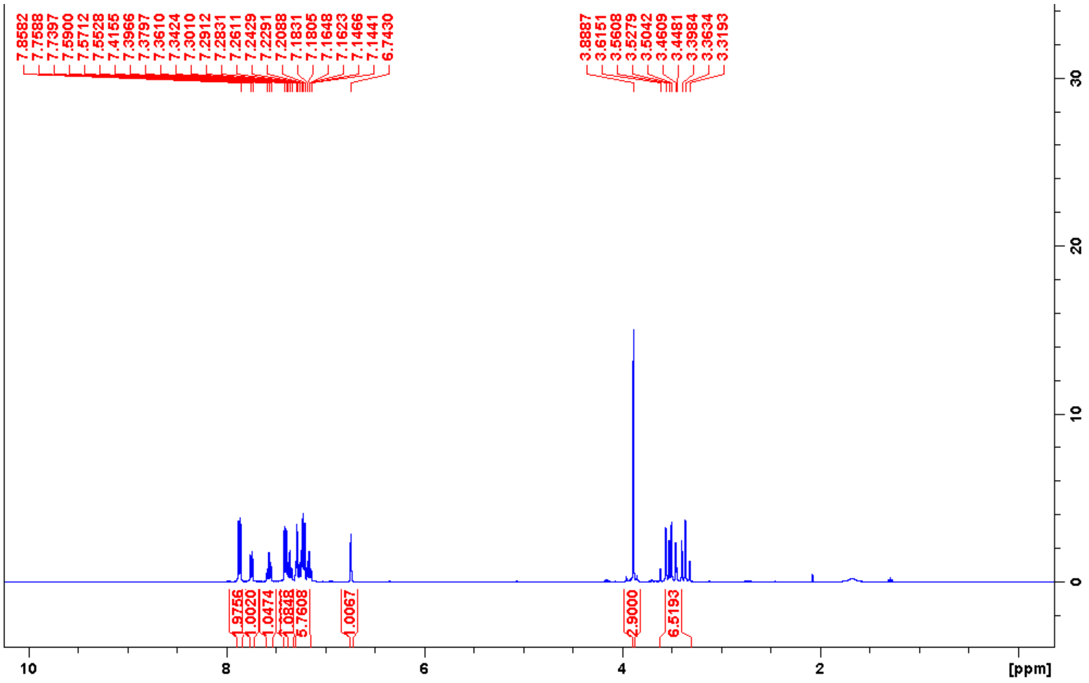

Figure S4. ^{1}H NMR spectrum of (*R*)-**4** synthesised from (*S*,*R*)-**8**.

(*S*)-4-((1'-oxo-1',3'-dihydro-1H,2'H-[2,2'-biinden]-2'-yl)methyl)benzoic acid (*S*)-10

Aqueous NaOH (30%, 0.67 g, 5.0 mmol, 8.0 eq.) was added to a stirred solution of (*S*)-**4** (0.25 g, 0.63 mmol, 1.0 eq.) in MeOH (2.0 mL). The resulting mixture was heated at 60 °C for 3 h. Water (15 mL) was added and the mixture was washed with DCM (2 x 10 mL). The aqueous layer was acidified with aqueous HCl (10%) and extracted with DCM (2 x 10 mL). The combined organic extracts were washed with brine (10 mL) and dried under reduced pressure yielding crude (*S*)-**10** acid (0.26 g) as an orange viscous oil. Purification by flash chromatography using 1:1 ethyl acetate:cyclohexane as eluent gave (*S*)-**10** (0.17 g, 98.8% ee) as a pale yellow solid.

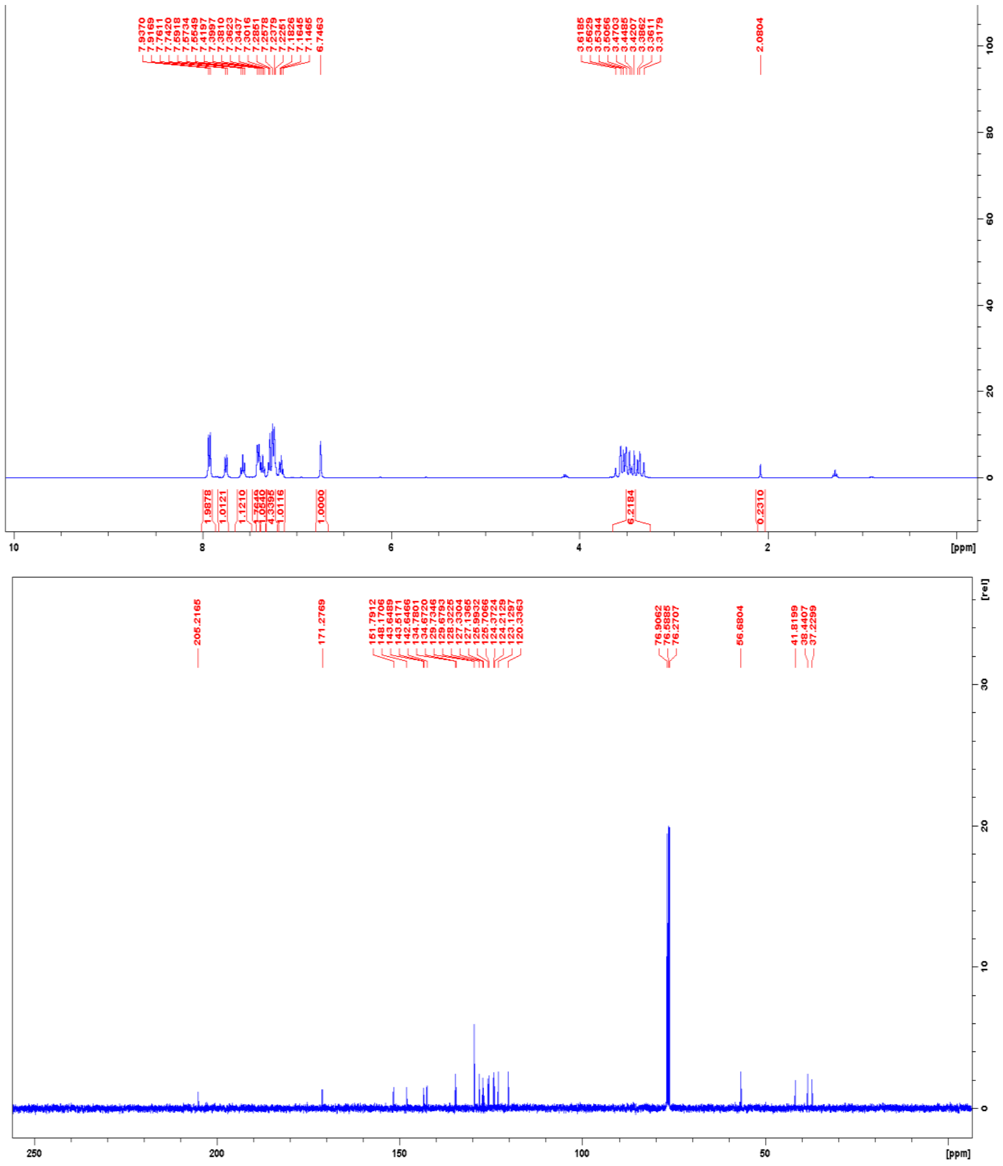

Figure S5. ^{1}H (upper) and ^{13}C (lower) NMR spectra of (*S*)-**10** synthesised from (*S*)-**4**.

(*R*)-4-((1'-oxo-1',3'-dihydro-1H,2'H-[2,2'-biinden]-2'-yl)methyl)benzoic acid (*R*)-10

Aqueous NaOH (30%, 0.67 g, 5.0 mmol, 8.0 eq.) was added to a stirred solution of (*R*)-**4** (0.25 g, 0.63 mmol, 1.0 eq.) in MeOH (2.0 mL). The resulting mixture was heated at 60 °C for 3 h. Water (15 mL) was added and the mixture was washed with DCM (2 x 10 mL). The aqueous layer was acidified with aqueous HCl (10%) and extracted with DCM (2 x 10 mL). The combined organic extracts were washed with brine (10 mL) and dried *in vacuo* yielding crude (*R*)-**10** (0.19 g) as a yellow solid. Purification by flash chromatography using 1:1 ethyl acetate:cyclohexane as eluent gave (*R*)-**10** (0.15 g, 95.7% ee) as a white solid.

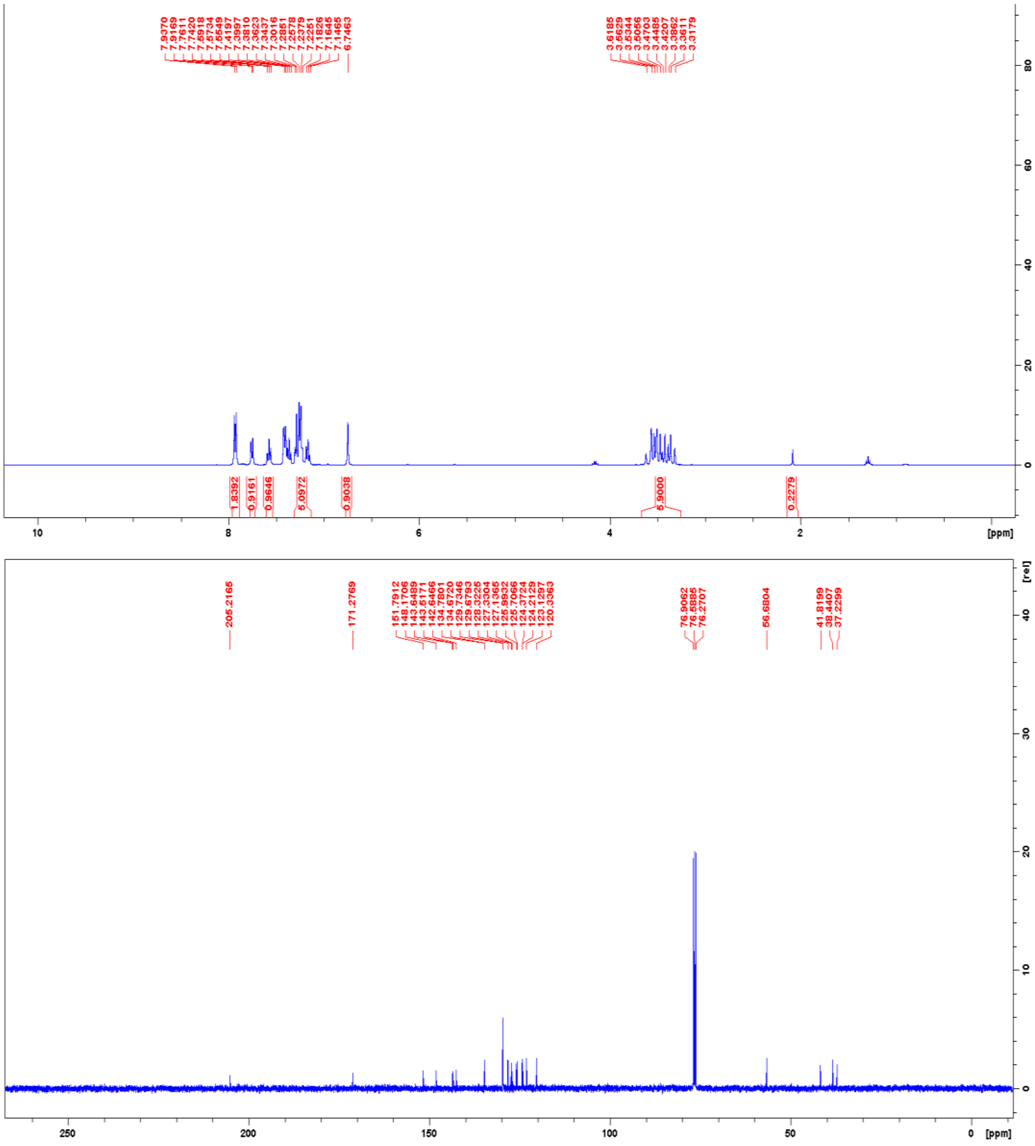

Figure S6. ^{1}H (upper) and ^{13}C (lower) NMR spectra of (*R*)-**10** synthesised from (*R*)-**4**.

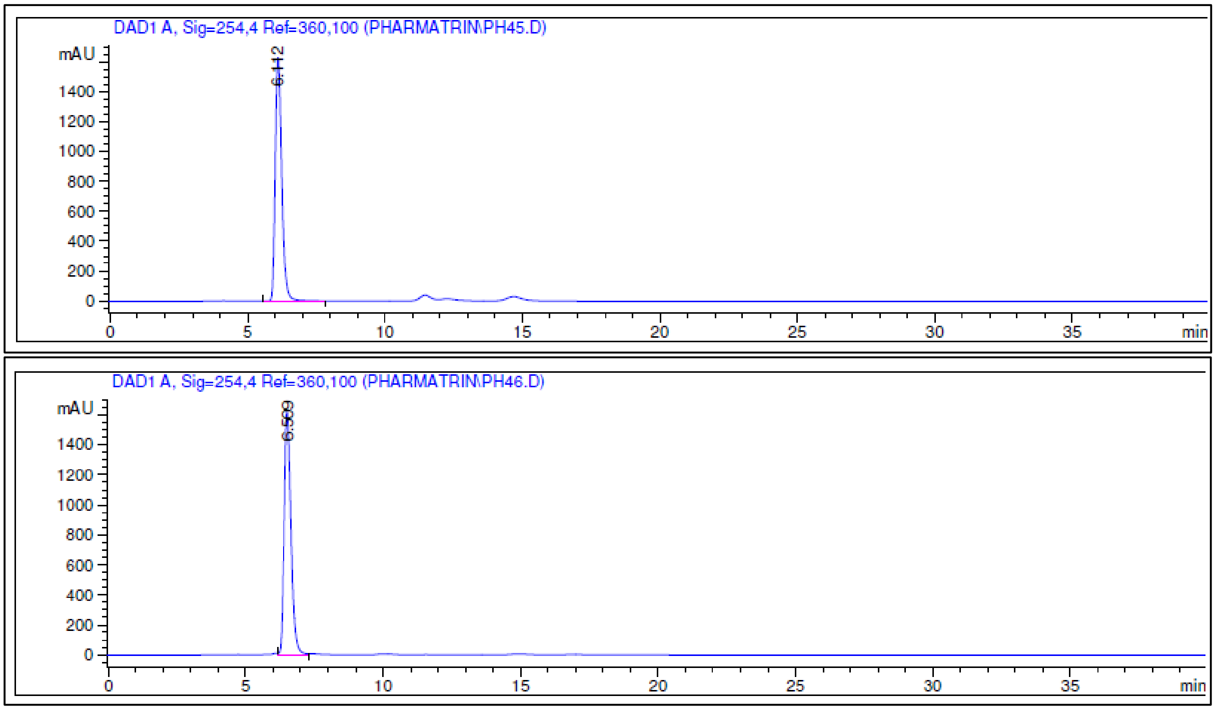

Figure S7. Chiral HPLC chromatograms of (*R*,*R*)-**7** (upper, RT=6.1 min) and (*S*,*S*)-**7** (lower, RT=6.5 min)

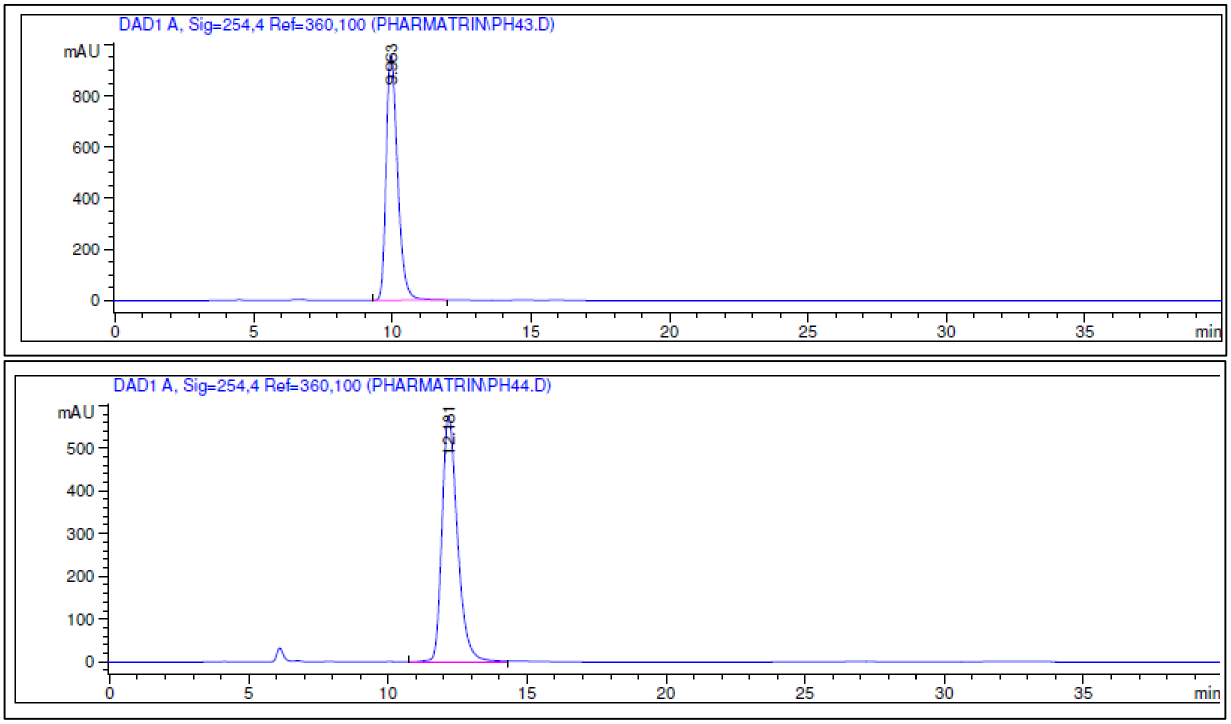

Figure S8. Chiral HPLC chromatograms of (*R*,*S*)-**8** (upper, RT=10.0 min) and (*S*,*R*)-**8** (lower, RT=12.2 min).

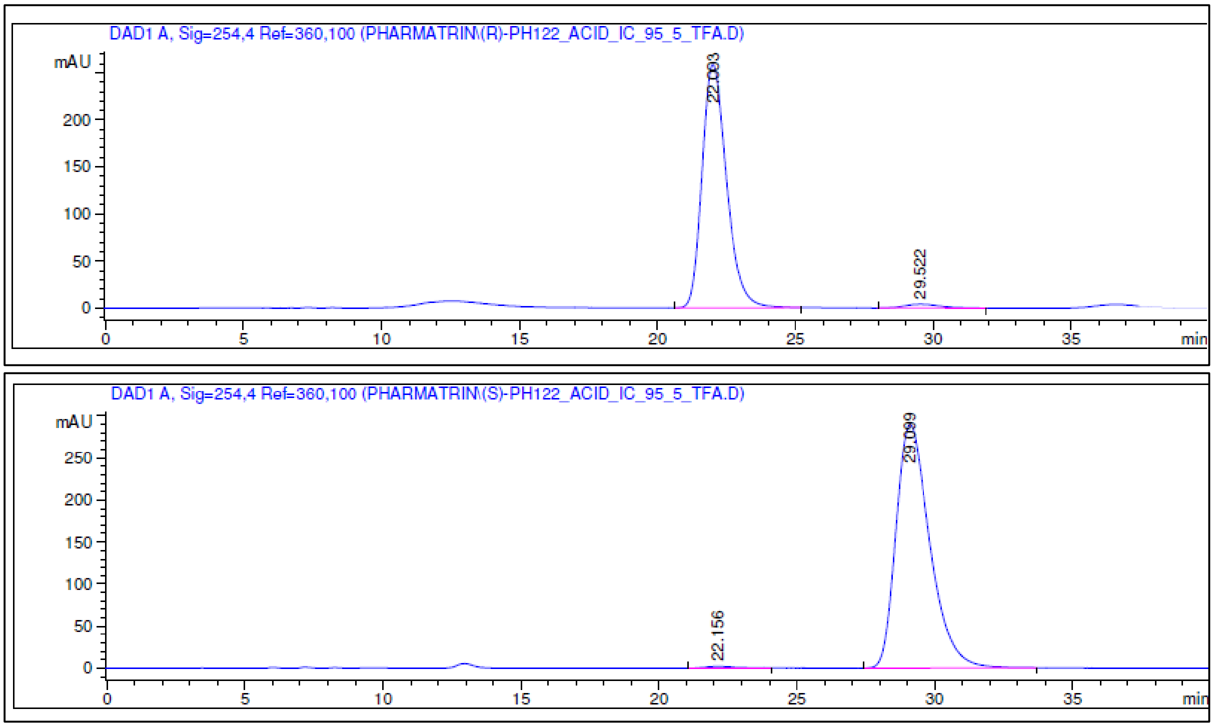

Figure S9. Chiral HPLC chromatograms of (*R*)-**10** (upper, RT=22.0 min) and (*S*)-**10** (lower, RT=29.1 min).

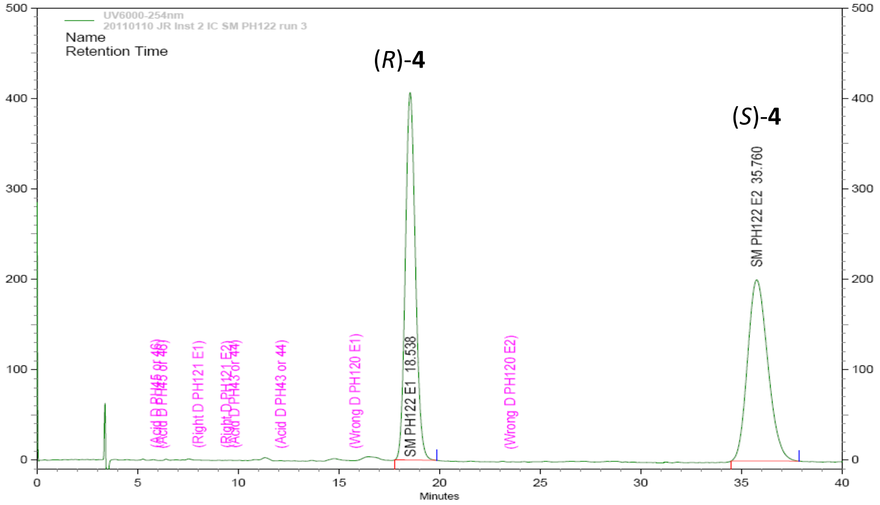

Figure S10. Chiral HPLC chromatogram of (*R*)-**4** & (*S*)-**4**.

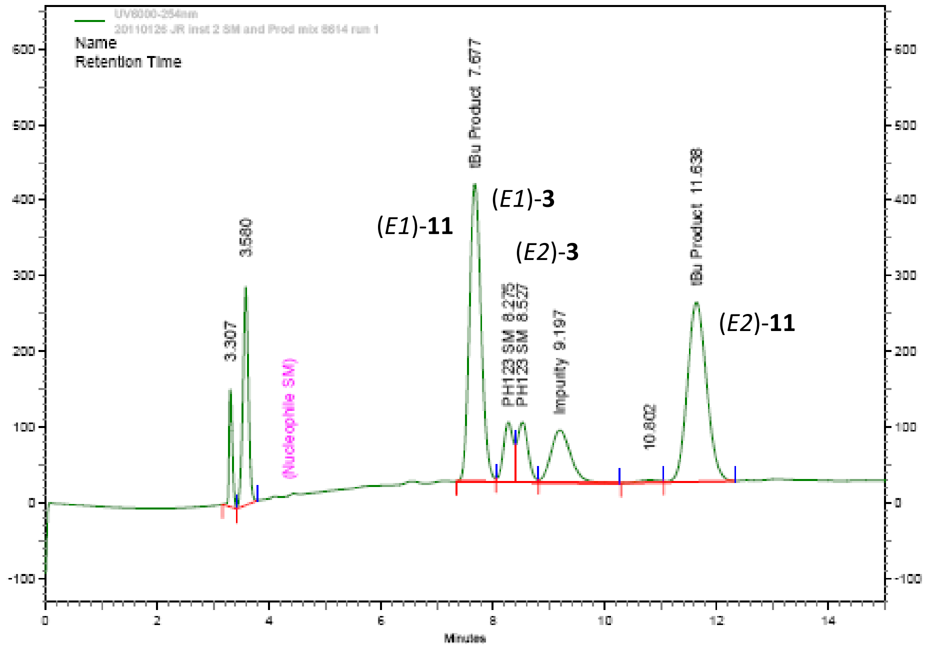

Figure S11. Chiral HPLC chromatogram of compounds **3** & **11**.

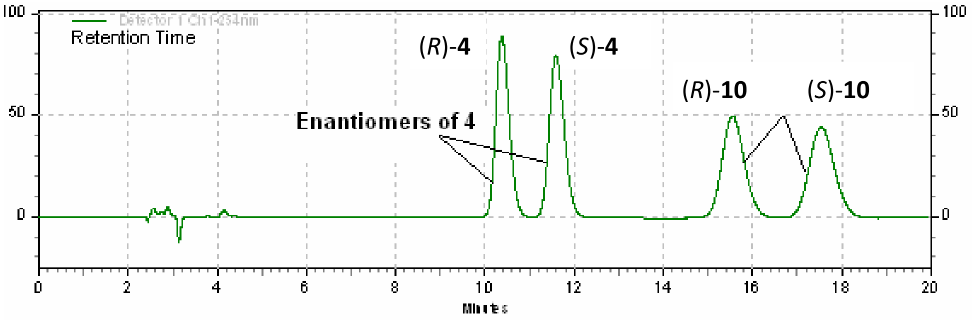

Figure S12. Chiral HPLC chromatogram of (*R*)-**4**, (*S*)-**4**, (*R*)-**10** & (*S*)-**10** for hydrolase screening.
